# Four New Flavan-3-ol Derivatives with Potent *α*-Glucosidase Inhibitory Activity from Black Tea Produced from *Camellia taliensis*

**DOI:** 10.3390/foods15091609

**Published:** 2026-05-06

**Authors:** Min Chen, Na Li, Jia-Huan Shang, Hong-Tao Zhu, Ying-Jun Zhang

**Affiliations:** 1 State Key Laboratory of Phytochemistry & Natural Medicines, Kunming Institute of Botany, Chinese Academy of Sciences, Kunming 650201, China; chenmin@mail.kib.ac.cn (M.C.); shangjiahuan@mail.kib.ac.cn (J.-H.S.); 2 University of Chinese Academy of Sciences, Beijing 100049, China; 3 Key Laboratory of Phytochemistry and Plant Resources in West China, Kunming Institute of Botany, Chinese Academy of Sciences, Kunming 650201, China; zhuhongtao@mail.kib.ac.cn

**Keywords:** *Camellia taliensis*, black tea, flavoalkaloids, flavan-3-ols, degradation products, *α*-glucosidase inhibitory activity

## Abstract

Black tea, a tea fully fermented by polyphenol oxidase, is widely recognized for its distinctive flavor and diverse health benefits largely attributed to its various phenolic components. An extensive phytochemical investigation of black tea made from the leaves of *Camellia taliensis* (Theaceae), a wild tea plant distributed in Yunnan province, China, led to the isolation and characterization of 15 flavan-3-ol derivatives, among which four previously unreported products (**1**–**4**), including two flavoalkaloids (**1**–**2**), one flavan-3-ol (**3**) and one nitrogen-containing phenol (**4**), were identified. When evaluated for *α*-glucosidase inhibitory activity, 10 of the isolates showed stronger activities than quercetin and acarbose (IC_50_ = 5.75 and 223.30 µM, respectively), with IC_50_ values ranging from 0.09 to 3.57 µM. Notably, compound **15** displayed exceptional potency with an IC_50_ of 0.09 μM, approximately 60-fold lower than that of quercetin. These findings highlight the potential of black tea produced from *C. taliensis* for the development of functional foods targeting postprandial hyperglycemia management.

## 1. Introduction

Tea, one of the most popular beverages, is classified into green, white, yellow, oolong, black and dark teas based on its different processing techniques [1]. Among them, black tea, a fully fermented tea by polyphenol oxidase, has attracted considerable attention from both consumers and researchers, due to its health benefits such as anti-aging, anti-diabetic, anti-hyperlipidemic and anti-cancer effects largely associated with its tea polyphenols comprising flavan-3-ols and their derivatives, flavonols and their glycosides as well as hydrolyzable tannins [2,3,4,5].

In black tea, flavan-3-ol monomers were primarily transformed into dimers (e.g., theaflavins and theasinensins), trimers (e.g., theadibenzotropolones), tetramers (e.g., bistheaflavins and theatribenzotropolones) and, ultimately, thearubigins, through catalyzed oxidation by polyphenol oxidase during the fermentation process [6,7,8,9,10]. Notably, a degradation product of flavan-3-ol, (3*R*)-2,5,7-trihydroxy-chroman-3-yl 3,4,5-trihydroxy-benzoate with the absence of the B-ring, was isolated from black tea in a previous study [11]. This finding suggested the presence of degradation reactions during processing in black tea.

A novel type of flavan-3-ol derivatives featuring a nitrogen-containing five-membered ring moiety on the A-ring, a subgroup of flavoalkaloids, has been reported in various teas (black, dark, white and green tea) and has recently garnered significant attention in tea chemistry [12,13,14,15]. Up to date, 10 flavoalkaloids with significant inhibitory activity against *α*-glucosidase and acetylcholinesterase have been isolated and identified from black tea [15,16].

*Camellia taliensis* (W. W. Smith) Melchior, a member of the genus *Camellia* sect. Thea., is a valuable wild germplasm resource and an important wild relative of the cultivated *C. sinensis* and *C. sinensis* var. *assamica* [17]. Its natural range extends from northern Myanmar to western and southwestern Yunnan Province, China, with isolated populations occurring along the Ailao Mountain range and the Lancang (Mekong) and Nujiang (Salween) river basins. Although the overall chemical profile of *C. taliensis* resembles that of cultivated tea—all being rich in flavan-3-ols and caffeine—*C. taliensis* typically contains slightly lower levels of these components. A distinguishing chemical feature of *C. taliensis* is its abundance of hydrolyzable tannins. Notably, the marker compound, 1,2-di-*O*-galloyl-4,6-*O*-(*S*)-hexahydroxydiphenoyl-*β*-D-glucopyranose, can account for up to 2.44% of the dry leaf weight [5]. Elucidating its chemical compositions may contribute to the more efficient utilization of its resources. Our earlier work on black tea derived from *C. taliensis* afforded 32 phenolic compounds, including hydrolyzable tannins, flavonols and glycosides, and simple phenolics, with notable antioxidant and *α*-glucosidase inhibitory activities [5]. However, the systematic investigation on flavan-3-ols and their derivatives present in black tea prepared from *C. taliensis* remains lacking. Moreover, the degradation product of flavan-3-ols has rarely been reported from black tea. In the present study, four previously undescribed degradation products of flavan-3-ols (**1**–**4**) were isolated and identified from black tea prepared from *C. taliensis*, together with 11 known flavan-3-ols and their derivatives. Furthermore, 10 of these isolates exhibited significant α-glucosidase inhibitory activity, and a preliminary analysis was conducted to evaluate the contribution of substituent groups (the *N*-ethyl-2-pyrrolidinone group, B-ring and galloyl group) to the inhibitory potency. The result provides a new insight into the chemical diversity and α-glucosidase inhibitory activity of black tea from *C. taliensis*. Herein, we report this study.

## 2. Materials and Methods

### 2.1. General Procedure

1D and 2D NMR spectra were recorded on Bruker NMR spectrometers (Bruker Corporation, Karlsruhe, Germany) operating at 600 and 500 MHz. Methanol-*d*4 (CD_3_OD) served as the solvent, with TMS as an internal standard. ESIMS and HRESIMS analyses were performed using a Shimadzu LCMS-2020 instrument (Shimadzu Corporation, Tokyo, Japan). Semi-HPLC was executed on a Hanbon HPLC system (Hanbon Sci. & Tech., Huai’an, China), which was fitted with a Thermo Hypersil GOLD aQ column (10 × 250 mm inner diameter, 5 µm). HPLC analysis was conducted using an Agilent 1290 (Agilent Technologies Inc., Santa Clara, CA, USA) fitted with DAD detection and a COSMOSIL C-18 column (4.6 × 250 mm inner diameter, 5 µm). UV absorption spectra were captured with the aid of a Shimadzu UV2401A spectrophotometer (Shimadzu Co., Kyoto, Japan), employing methanol as the solvent. Optical rotation values in methanol were examined using a JASCO P-1020 polarimeter (JASCO, Tokyo, Japan). The electronic circular dichroism data were acquired utilizing a Chirascan V100 spectrometer (Applied Photophysics Ltd., Leatherhead, Surrey, UK). Absorbance was recorded using a Tecan Spark multimode microplate reader with SparkControl v2.3 software (Tecan Group Ltd., Männedorf, Switzerland).

### 2.2. Chemicals and Reagents

Column chromatography (CC) was carried out utilizing a variety of stationary phases, including Sephadex LH-20 (25–100 µm) (GE Healthcare Bio-Science AB, Uppsala, Sweden), Toyopearl HW-40F (37–70 µm) (GE Healthcare Bio-Science AB, Uppsala, Sweden), Diaion HP20SS (63–150 µm) (Mitsubishi Chemical Corporation, Tokyo, Japan), MCI-gel CHP20P (75–100 µm) (Mitsubishi Chemical Corporation, Tokyo, Japan), and RP-18 (40–60 µm, Merck, Darmstadt, Germany). Thin-layer chromatography (TLC) was performed on precoated silica gel F254 plates (Qingdao Haiyang Chemical Co., Ltd., Qingdao, China). The elution was performed using mixtures of toluene, ethyl formate, and formic acid (Shanghai Titan Scientific Co., Ltd., Shanghai, China) in the volume ratios of 1:7:2, 1:7:1, and 2:7:1. The spot was visualized under UV radiation at 254 nm and then dipping with mixtures of sulfuric acid and ethanol (1:9, *v*/*v*), followed by heating. The deionized water and redistilled organic solvents such as methanol (MeOH), acetone, ethanol (EtOH), ethyl acetate (EtOAc), chloroform (CHCl_3_), and *n*-butanol (*n*-BuOH) were employed for isolation and purification. HPLC was carried out using chromatography-grade acetonitrile (MeCN), formic acid (HCOOH), and deionized water. CD_3_OD with 0.03% TMS (Shanghai Titan Co. Ltd., Shanghai, China) was used as a solvent in NMR spectroscopy. Phloroglucinol was purchased from Sichuan Weikeqi Biological Technology Co., Ltd. (Sichuan, China). The α-glucosidase, 4-nitrophenol-*α*-D-glucopyranoside (PNPG) and phosphate buffer were purchased from Shanghai Yuanye Bio-Technology Co., Ltd. (Shanghai, China). Dimethyl sulfoxide (DMSO) was purchased from Shanghai Titan Scientific Co., Ltd. (Shanghai, China).

### 2.3. Materials

The black tea investigated in this work was manufactured from leaves of *C. taliensis* harvested in Dasi township, Fengqing County, Yunnan Province, China, in 2021. The voucher specimen (KIB-Z-2107B17) has been deposited at the Key Laboratory of Phytochemistry and Plant Resources in West China, Kunming Institute of Botany (KIB), Chinese Academy of Sciences (CAS).

### 2.4. Extraction and Isolation

Black tea derived from *C. taliensis* (10.2 kg) was subjected to four successive extractions with 60% aqueous acetone (40 L each, one week per extraction) at room temperature. Following removal of acetone under reduced pressure, the resulting crude extract (2.37 kg, yield 23.2%) was taken up in water and then fractionated using a sequence of solvents, CHCl_3_, EtOAc, and *n*-BuOH. The EtOAc fraction (350 g, representing 14.7% of the crude extract) was then fractionated over Sephadex LH-20 CC by elution with aqueous MeOH (0–100%), yielding six fractions (Fr. 1–Fr. 6).

Fr. 3 (121.2 g) was processed through Diaion HP20SS CC (h: 60 cm, d: 7 cm), with aqueous MeOH (0–100%) as the mobile phase, to result in the separation of 15 subfractions (Fr. 3-1–Fr. 3-15). Following that, Fr. 3-6 (10.41 g) and Fr. 3-7 (16.93 g) were each passed through MCI-gel CHP20P CC using a gradient of aqueous MeOH (0–100%), to yield seven subfractions (Fr. 3-6-1–Fr. 3-6-7) and 10 subfractions (Fr. 3-7-1–Fr. 3-7-10), respectively. Toyopearl HW-40F and Sephadex LH-20 CC (aqueous MeOH, 0–100%), and further semi-HPLC (aqueous MeCN with 1.5‰ HCOOH, 15%), were employed to gain **1** (3.3 mg), **2** (3.0 mg) and **5** (8.9 mg) from Fr. 3-7-3 (4.16 g), and **8** (7.0 mg) from Fr. 3-7-2 (1.18 g). Fr. 3-7-4 (4.12 g) was purified by repeated CC over Toyopearl HW-40F, RP-18 and Sephadex LH-20 (aqueous MeOH, 0–100%), followed by semi-HPLC (aqueous MeCN, 21% and 15%), to afford **9** (2.1 mg) and **10** (10.9 mg). Repeated CC over Toyopearl HW-40F, MCI-gel CHP20P and Sephadex LH-20 (aqueous MeOH, 0–100%), followed with semi-HPLC (aqueous MeCN with 1.5‰ HCOOH, 10%, 14% and 19%), afforded **3** (5.9 mg), **6** (10.3 mg) and **7** (4.1 mg) from Fr. 3-2 (5.14 g), and **12** (7.2 mg) from Fr. 3-6 (10.41 g). Similarly, Fr. 5 (47.7 g) was subjected to CC on Diaion HP20SS (h: 60 cm, d: 7 cm) to give nine subfractions (Fr. 5-1–Fr. 5-9). Among them, Fr. 5-4 (8.04 g) was treated using Toyopearl HW-40F CC (aqueous MeOH, 0–100%) to afford 11 subfractions (Fr. 5-4-1–Fr. 5-4-11). Then, Sephadex LH-20 CC (aqueous MeOH, 0–100%) and further semi-HPLC (aqueous MeCN, 16% and 28%) afforded **11** (1.4 mg) from Fr. 5-4-6 (91.7 mg), and **13** (7.2 mg) and **14** (2.1 mg) from Fr. 5-4-10 (150.3 mg). Fr. 5-4-11 (1.14 g) was subjected to CC on Sephadex LH-20 (aqueous MeOH 0–100%) to yield **15** (0.28 g). Fr. 2 (32.2 g) was treated with multiple CC over MCI-gel CHP20P and Sephadex LH-20 to give Fr. 2-8-9 (34.3 mg), which was purified through semi-HPLC (aqueous MeCN, 15%) to give **4** (2.5 mg).

### 2.5. α-Glucosidase Inhibitory Assay

An enzyme–inhibitor screening model using PNPG as substrate was employed to assess the inhibitory activity of isolates on *α*-glucosidase, as recorded in our prior paper [5]. Briefly, *α*-glucosidase and PNPG were dissolved in phosphate buffer (pH 7.0). Test samples were dissolved in DMSO. The assay was performed in a 96-well microplate by successively adding 1 μL sample solution (final concentration of 50 μM), 50 μL α-glucosidase solution (final concentration 0.025 U/mL), 109 μL phosphate buffer, and 40 μL PNPG solution (final concentration 1 mM) to a total volume of 200 μL per well. All assays were performed in triplicate (*n* = 3). Blank controls (without enzyme) and positive controls (quercetin and acarbose) were included in each plate. After incubation at 37 °C for 50 min, the OD value was measured at 405 nm using a microplate reader. The inhibition ratio was calculated according to the following formula: inhibition ratio (%) = (OD_blank_ − OD_test_)/OD_blank_ × 100%, and IC_50_ values were determined according to the Reed–Muench method [18].

### 2.6. Statistical Analysis

All experiments were performed in triplicate (*n* = 3), and data are expressed as means ± standard deviation (SD). The distribution of the replicate data was confirmed to follow a normal distribution. Homogeneity of variances was assessed using the *F*-test. Statistical comparisons of IC_50_ values between compounds were performed using Student’s *t*-test. *p* < 0.05 was considered statistically significant.

## 3. Results and Discussion

### 3.1. Identification of Compounds ***1**–**15***

**Figure 1:**
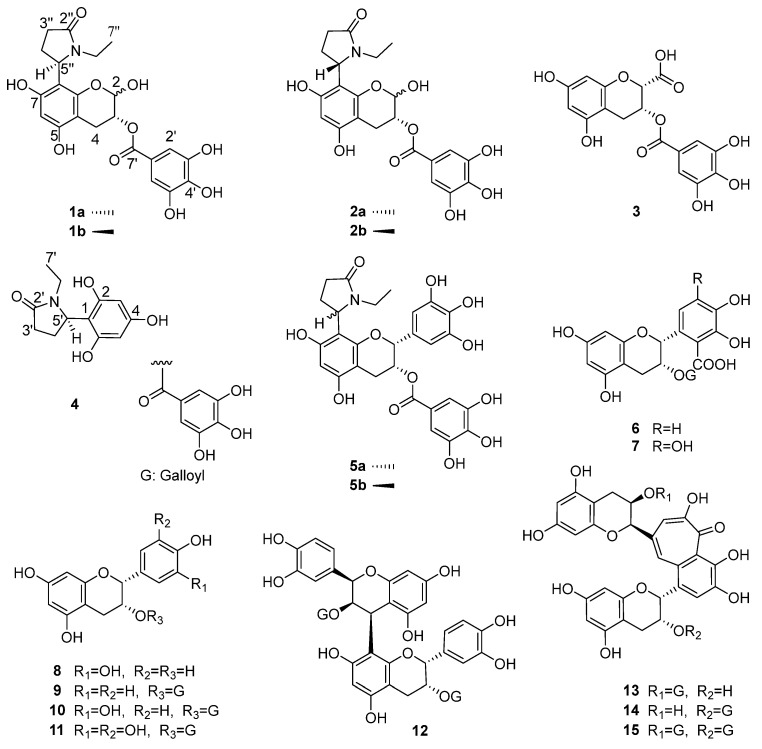
Compounds **1**–**15** isolated from black tea produced from *C. taliensis*.

The 60% aqueous acetone extract of black tea from *C. taliensis* was dissolved in water and sequentially partitioned with CHCl_3_, EtOAc, and *n*-BuOH. Subsequent purification of the EtOAc fraction through multiple CC and semipreparative HPLC yielded four new degradation products, including two flavoalkaloids (**1**–**2**), one flavan-3-ol (**3**) and one nitrogen-containing compound (**4**). In addition, 11 known compounds (Figure 1) were obtained and identified as flavoalkaloids, (−)-8-(5″*R*)-*N*-ethyl-2-pyrrolidinone epigallocatechin-3-*O*-gallate (**5a**) [16] and (−)-8-(5″*S*)-*N*-ethyl-2-pyrrolidinone epigallocatechin-3-*O*-gallate (**5b**) [16], and 10 flavan-3-ols, including theaflavoid A (**6**) [19], theaflavoid C (**7**) [19], (−)-epicatechin (**8**) [20], (−)-epiafzelechin 3-*O*-gallate (**9**) [21], (−)-epicatechin 3-*O*-gallate (**10**) [20], (−)-epigallocatechin 3-*O*-gallate (**11**) [20], procyanidin B2 3,3″-*O*-digallate (**12**) [22], theaflavin 3-gallate (**13**) [23], theaflavin 3’-gallate (**14**) [23] and theaflavin 3,3’-digallate (**15**) [23], through extensive spectroscopic analyses and comparison with the literature data. Among them, six compounds (**5**, **9**, **12**–**15**) were obtained from *C. taliensis* for the first time.

Compound **1**. Yellow-brown amorphous powder; ESI-MS: *m*/*z* 460 [M − H]^−^; HRESI-MS: *m*/*z* 460.1248 [M − H]^−^ (calculated for C_22_H_22_NO_10_, 460.1249); ${\left[\alpha \right]}_{D}^{20}$ − 56.02 (*c* 0.146, MeOH); UV *λ*_max_ (MeOH) (log *ε*): 277 (3.88), 210 (4.67) nm.

**1a** (2*R*,3*R*). ^1^H NMR (600 MHz, CD_3_OD): *δ* 6.88 (s, 2H, H-2′,6′), 6.01 (s, H-6), 5.42 (br.s, H-2), 5.38 (dd, *J* = 9.5, 5.6 Hz, H-5″), 5.07 (d, *J* = 3.4 Hz, H-3), 3.35–3.30 (m, H-6″a), 2.96–2.83 (m, H-4a), 2.78 (d, *J* = 17.4 Hz, H-4b), 2.66 (m, H-6″b), 2.64–2.56 (m, H, H-3″a), 2.44–2.35 (m, H-3″b), 2.29 (dtd, *J* = 16.0, 9.5, 5.2 Hz, H-4″a), 2.16 (dq, *J* = 16.0, 5.6 Hz, H-4″b), 0.98 (t, *J* = 7.2 Hz, 3H, 7″-CH_3_). ^13^C NMR (150 MHz, CD_3_OD): *δ* 177.6 (C-2″), 167.9 (C-7′), 157.0 (C-5), 156.1 (C-7), 153.0 (C-9), 146.4 (C-3′,5′), 139.9 (C-4′), 121.2 (C-1′), 110.2 (C-2′,6′), 106.0 (C-8), 100.1 (C-10), 96.0 (C-6), 91.1 (C-2), 68.1 (C-3), 54.3 (C-5″), 36.6 (C-6″), 32.6 (C-3″), 24.6 (C-4″), 21.1 (C-4), 12.7 (C-7″).

**1b** (2*S*,3*R*). ^1^H NMR (600 MHz, CD_3_OD): *δ* 7.06 (m, 2H, H-2′,6′), 5.94 (s, H-6), 5.48 (br.s, H-2), 5.38 (dd, *J* = 9.5, 5.6 Hz, H-5″), 5.13 (d, *J* = 2.7 Hz, H-3), 3.35–3.30 (m, H-6″a), 2.96–2.83 (m, H-4a), 2.78 (d, *J* = 17.4 Hz, H-4b), 2.66 (m, H-6″b), 2.64–2.56 (m, H, H-3″a), 2.44–2.35 (m, H-3″b), 2.29 (dtd, *J* = 16.0, 9.5, 5.2 Hz, H-4″a), 2.16 (dq, *J* = 16.0, 5.6 Hz, H-4″b), 0.91 (t, *J* = 7.4 Hz, 3H, 7″-CH_3_). ^13^C NMR (150 MHz, CD_3_OD): *δ* 177.6 (C-2″), 167.6 (C-7′), 157.0 (C-5), 156.1 (C-7), 153.0 (C-9), 146.5 (C-3′,5′), 140.0 (C-4′), 121.3 (C-1′), 110.3 (C-2′,6′), 106.0 (C-8), 100.1 (C-10), 96.9 (C-6), 91.6 (C-2), 68.4 (C-3), 54.1 (C-5″), 36.4 (C-6″), 32.6 (C-3″), 24.7 (C-4″), 21.1 (C-4), 12.6 (C-7″).

Compound **2**. Yellow-brown amorphous powder; ESI-MS: *m*/*z* 460 [M − H]^−^; HRESI-MS: *m*/*z* 460.1246 [M − H]^−^ (calculated for C_22_H_22_NO_10_, 460.1249); ${\left[\alpha \right]}_{D}^{19}$ + 2.80 (*c* 0.065, MeOH); UV *λ*_max_ (MeOH) (log *ε*): 278 (3.86), 210 (4.72) nm.

**2a** (2*R*,3*R*). ^1^H NMR (600 MHz, CD_3_OD): *δ* 6.93 (s, 2H, H-2′,6′), 6.02 (s, H-6), 5.42 (dd, *J* = 9.4, 5.2 Hz, H-5″), 5.60–5.48 (m, H-2), 5.14 (dd, *J* = 4.5, 2.6 Hz, H-3), 3.56–3.40 (m, H-6″a), 2.99–2.86 (br.d, *J* = 17.2 Hz, H-4a), 2.85–2.73 (br.d, *J* = 17.2 Hz, H-4b), 2.68 (dd, *J* = 15.3, 8.5 Hz, H-3″a), 2.62 (dq, *J* = 14.2, 7.6 Hz, H-6″b), 2.41 (t, *J* = 9.1 Hz, H-3″b), 2.35–2.24 (m, H-4″a), 2.18 (dd, *J* = 9.4, 6.0 Hz, H-4″b), 1.00 (t, *J* = 7.6 Hz, 3H, 7″-CH_3_). ^13^C NMR (150 MHz, CD_3_OD): *δ* 177.6 (C-2″), 167.7 (C-7′), 157.0 (C-7), 156.8 (C-5), 153.0 (C-9), 146.4 (C-3′,5′), 140.0 (C-4′), 121.3 (C-1′), 110.1 (C-2′,6′), 105.9 (C-8), 99.1 (C-10), 96.1 (C-6), 91.2 (C-2), 68.1 (C-3), 54.0 (C-5″), 36.3 (C-6″), 32.6 (C-3″), 24.5 (C-4″), 21.3 (C-4), 12.5 (C-7″).

**2b** (2*S*,3*R*). ^1^H NMR (600 MHz, CD_3_OD): *δ* 7.09 (s, 2H, H-2′,6′), 5.96 (s, H-6), 5.42 (dd, *J* = 9.4, 5.2 Hz, H-5″), 5.40 (d, *J* = 4.3 Hz, H-2), 5.09 (m, H-3), 3.56–3.40 (m, H-6″a), 2.99–2.86 (br.d, *J* = 17.2 Hz, H-4a), 2.85–2.73 (br.d, *J* = 17.2 Hz, H-4b), 2.68 (dd, *J* = 15.3, 8.5 Hz, H-3″a), 2.62 (dq, *J* = 14.2, 7.3 Hz, H-6″b), 2.41 (t, *J* = 9.1 Hz, H-3″b), 2.35–2.24 (m, H-4″a), 2.18 (dd, *J* = 9.4, 6.0 Hz, H-4″b), 0.89 (t, *J* = 7.3 Hz, 3H, 7″-CH_3_). ^13^C NMR (150 MHz, CD_3_OD): *δ* 178.0 (C-2″), 167.7 (C-7′), 157.0 (C-7), 156.8 (C-5), 153.0 (C-9), 146.5 (C-3′,5′), 140.0 (C-4′), 121.3 (C-1′), 110.3 (C-2′,6′), 105.9 (C-8), 99.1 (C-10), 96.9 (C-6), 91.2 (C-2), 69.5 (C-3), 53.8 (C-5″), 36.5 (C-6″), 32.6 (C-3″), 24.5 (C-4″), 21.3 (C-4), 12.6 (C-7″).

Compound **3**. Brown amorphous powder; ESI-MS: *m*/*z* 377 [M − H]^−^; HRESI-MS: *m*/*z* 377.0511 [M − H]^−^ (calculated for C_17_H_13_O_10_, 377.0514); ${\left[\alpha \right]}_{D}^{20}$ − 39.18 (*c* 0.097, MeOH); UV *λ*_max_ (MeOH) (log *ε*): 278 (4.05), 207 (4.71) nm. ^1^H NMR (500 MHz, CD_3_OD): *δ* 6.98 (s, 2H, H-2′,6′), 6.02 (d, *J* = 2.4 Hz, H-8), 5.95 (d, *J* = 2.4 Hz, H-6), 5.81 (br.s, H-3), 4.69 (s, H-2), 2.99–2.87 (m, 2H, 4-CH_2_). ^13^C NMR (125 MHz, CD_3_OD): *δ* 172.7 (COOH), 167.5 (C-7′), 158.0 (C-7), 157.8 (C-5), 155.6 (C-9), 146.4 (C-3′,5′), 140.0 (C-4′), 121.2 (C-1′), 110.2 (C-2′,6′), 99.0 (C-10), 96.9 (C-6), 96.0 (C-8), 75.9 (C-2), 68.1 (C-3), 26.3 (C-4).

Compound **4**. Yellow-green amorphous powder; ESI-MS: *m*/*z* 236 [M − H]^−^; HRESI-MS: *m*/*z* 236.0926 [M − H]^−^ (calculated for C_12_H_14_NO_4_, 236.0928); ${\left[\alpha \right]}_{D}^{23}$ − 23.4 (*c* 0.100, MeOH); UV *λ*_max_ (MeOH) (log *ε*): 209 (4.66) nm. ^1^H NMR (600 MHz, CD_3_OD): *δ* 5.83 (s, 2H, H-3,5), 5.36 (dd, *J* = 9.6, 5.6 Hz, H-5′), 3.45 (dq, *J* = 14.5, 7.2 Hz, H-6′a), 2.65 (ddd, *J* =14.5, 7.0, 6.8 Hz, H-6′b), 2.62 (m, H-3′a), 2.39 (ddd, *J* = 16.6, 10.7, 6.0 Hz, H-3′b), 2.33–2.24 (m, H-4′a), 2.16 (dq, *J* = 16.6, 5.6 Hz, H-4′b), 0.99 (t, *J* = 7.2 Hz, 7′-CH_3_). ^13^C NMR (150 MHz, CD_3_OD): *δ* 177.5 (C-2′), 159.4 (C-2,4,6), 105.3 (C-1), 96.0 (C-3), 95.0 (C-5), 54.1 (C-5′), 36.3 (C-6′), 32.6 (C-3′), 24.4 (C-4′), 12.6 (C-7′).

**Figure 2:**
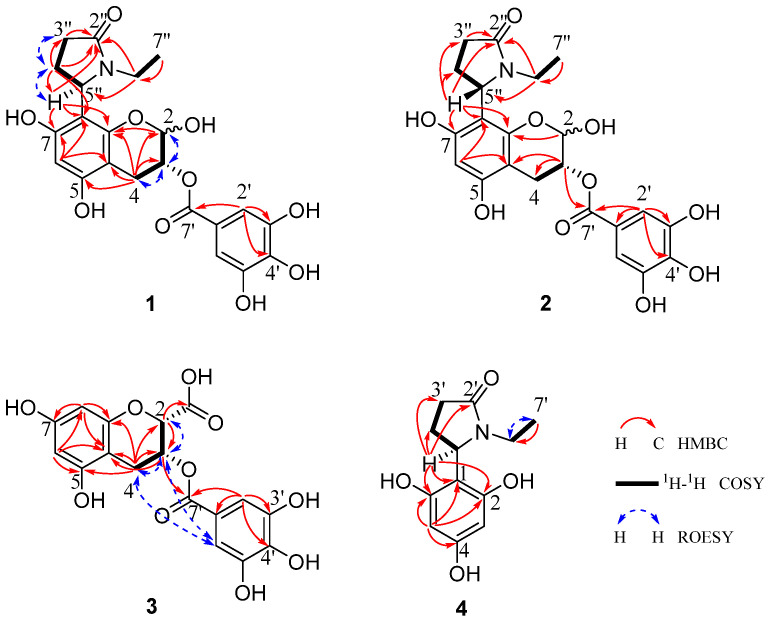
Key 2D NMR correlations of compounds **1**–**4**.

Compounds **1a** and **1b**, obtained as a yellow-brown amorphous powder with ${\left[\alpha \right]}_{D}^{20}$ − 56.02 (*c* 0.146, MeOH) (Figure S9), could not be separated, even after repeated purification attempts. The molecular formula, C_22_H_23_NO_10_, was deduced from HRESI-MS at *m*/*z* 460.1248 [M − H]^−^ (calculated for 460.1249) (Figure S7), demonstrating 12 degrees of unsaturation. The structure of compound **1** was determined to be a flavan-3-ol derivative lacking the B-ring but with an additional hydroxyl group, as suggested by the marked downfield shift of C-2 (*δ*_C_ 91.1) compared to **5**, the absence of B-ring signals, and the molecular formula [24]. Meanwhile, compound **1** with a free hydroxyl group at C-2 was also confirmed through three pairs of signals [H-2 (*δ* 5.42 and 5.48, each br.s), C-2 (*δ* 91.1 and 91.6); H-3 (*δ* 5.07 and 5.13, each d), C-3 (*δ* 68.1 and 68.4); H-2′/6′ (*δ* 6.88 and 7.06, each s), C-2′/6′ (*δ* 110.2 and 110.3)], which were very similar to those of (3*R*)-2,5,7-trihydroxychroman-3-yl 3,4,5-trihydroxybenzoate, a flavan-3-ol derivative with a free hydroxyl group at C-2 isolated from black tea [11]. The galloyl group was deduced by the characteristic two-proton singlet (*δ*_H_ 6.88, 2H, s, H-2′,6′) in the ^1^H NMR and seven sp^2^ carbon signals [*δ*_C_ 146.4 (C-3′,5′), 139.9 (C-4′), 121.2 (C-1′), 110.2 (C-2′,6′), 167.9 (C-7′)] in the ^13^C NMR spectra, with further support from the corresponding mass spectrometric fragment ions (*m*/*z* 460 [M − H]^−^, 290 [M − H−gallic acid]^−^). The existence of one *N*-ethyl-2-pyrrolidinone group was deduced from the carbon signals [*δ*_C_ 177.6 (C-2″), 54.3 (C-5″), 36.6 (C-6″), 32.6 (C-3″), 24.6 (C-4″), 12.7 (C-7″)] observed in the ^13^C NMR spectrum of **1** (Table S1) and by correlating the NMR data of **5** [24]. Furthermore, the HMBC correlations (Figure 2) from H-5″ (*δ*_H_ 5.38) to C-8 (*δ*_C_ 106.0) revealed that the *N*-ethyl-2-pyrrolidinone group was connected at C-8. The different configurations of compounds **1a** and **1b** were inferred from the observation of two sets of proton and carbon signals in the ^1^H and ^13^C NMR spectra. Other 2D NMR correlations (Figure 2) also confirmed the above result. Thus, the planar structure of **1** was determined to be (3*R*)-8-(*N*-ethyl-2-pyrrolidinone)-2,5,7-trihydroxychroman-3-*O*-gallate, as shown in Figure 1.

Compounds **2a** and **2b**, obtained as a yellow-brown amorphous powder with ${\left[\alpha \right]}_{D}^{19}$ + 2.80 (*c* 0.065, MeOH) (Figure S17), are also an inseparable mixture. The molecular formula, C_22_H_23_NO_10_, was deduced from HRESI-MS at *m*/*z* 460.1246 [M − H]^−^ (calculated for 460.1249) (Figure S15), demonstrating 12 degrees of unsaturation. In a similar way, **2** was established to share the same planar structure as (3*R*)-8-(*N*-ethyl-2-pyrrolidinone)-2,5,7-trihydroxychroman-3-*O*-gallate, shown in Figure 1.

**Figure 3:**
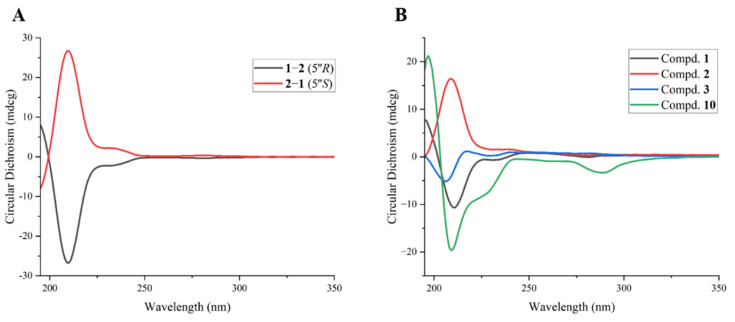
Subtractive CD curves of compounds **1** and **2** (**A**) and CD spectra of compounds **1**, **2**, **3** and **10** (**B**).

The absolute configuration at C-3 was proposed as *R* for both **1** and **2** according to the biosynthetic pathway. Each compound is a mixture of two epimers at C-2, denoted as **1a**/**1b** and **2a**/**2b**, respectively. Meanwhile, they themselves are a pair of epimers relative to each other at the C-5″ position. Previous studies pointed out that the absolute configuration at C-5″ can be deduced by subtracting the CD spectrum of one stereoisomer from another with the same C-2/C-3 configurations [15,24,25]. As a negative Cotton effect at 210 nm resulted from subtracting the CD curves of **2** from that of **1** (Figure 3A), compound **1** was suggested to possess the 5″*R*-configuration. Similarly, compound **2** was assigned as the 5″*S*-configuration (Figure 3A). On the basis of the above evidence, compounds **1** and **2** were established as (3*R*)-8-((5″*R*)-*N*-ethyl-2-pyrrolidinone)-2,5,7-trihydroxychroman-3-*O*-gallate and (3*R*)-8-((5″*S*)-*N*-ethyl-2-pyrrolidinone)-2,5,7-trihydroxychroman-3-*O*-gallate, respectively (Figure 1). Furthermore, the ^1^H and ^13^C NMR data for **1** and **2**, combined with the coupling constants of H-2 and H-3, allowed the signals to be respectively distinguished into two groups, identified as **1a**/**1b** and **2a**/**2b** (Tables S1 and S2, Figures S1 and S10).

Compound **3** was obtained as a brown amorphous powder with ${\left[\alpha \right]}_{D}^{20}$ − 39.18 (*c* 0.097, MeOH) (Figure S26). Its molecular formula, C_17_H_14_O_10_, was confirmed by HRESI-MS at *m*/*z* 377.0511 [M − H]^−^ (calculated for 377.0514) (Figure S24), proving 11 degrees of unsaturation. The ^1^H NMR spectrum of **3** (Table S3) revealed a series of proton signals [*δ*_H_ 5.81 (br.s, H-3), 4.69 (s, H-2), 2.99–2.87 (m, 4-CH_2_) (ring C), 6.02 (d, *J* = 2.4 Hz, H-8), 5.95 (d, *J* = 2.4 Hz, H-6) (ring A), 6.98 (s, H-2′,6′) (galloyl)] that closely resembled those of theaflavoid A (**6**), indicating the presence of a flavan-3-ol skeleton in the structure of **3**. However, the obvious up-field shift of H-2 (*δ*_H_ 4.69, s) in the flavan-3-ol skeleton, together with the absence of B-ring signals, suggested that compound **3** was a flavan-3-ol derivative containing only rings A and C. Additionally, the presence of a carboxyl group was inferred by the HMBC correlation from H-2 (*δ*_H_ 4.69) to a carbonyl carbon (*δ*_C_ 172.7), which was consistent with the molecular formula. The structure of compound **3** was thus established as a flavan-3-ol derivative lacking the B-ring but bearing an additional carboxyl group at C-2. This conclusion was further supported by comparing its similar 1D NMR data and different molecular formula with those of 3,4-dihydro-5,7-dihydroxy-2H-1-benzopyran-3-yl 3,4,5-trihydroxybenzoate [26]. Other 2D NMR correlations (Figure 2) also confirmed the planar structure of **3**. The proton signals of H-2 and H-3 manifested as a broad singlet or a doublet with a small coupling constant when the substituents at the C-2 and C-3 were in a *cis* configuration, as demonstrated in the ^1^H NMR spectra of theaflavoid A (**6**) and theaflavoid C (**7**) [19]. Thus, the C-2*S*, C-3*R* stereochemistry was inferred from the proton signals of H-2 (*δ*_H_ 4.69, s) and H-3 (*δ*_H_ 5.81, br.s), coupled with a CD curve similar to that of ECG (**10**) (Figure 3B) [19]. Finally, the structure of **3** was assigned as (2*S*,3*R*)-5,7-dihydroxy-3-((3,4,5-trihydroxybenzoyl)oxy)chromane-2-carboxylic acid (Figure 1).

**Figure 4:**
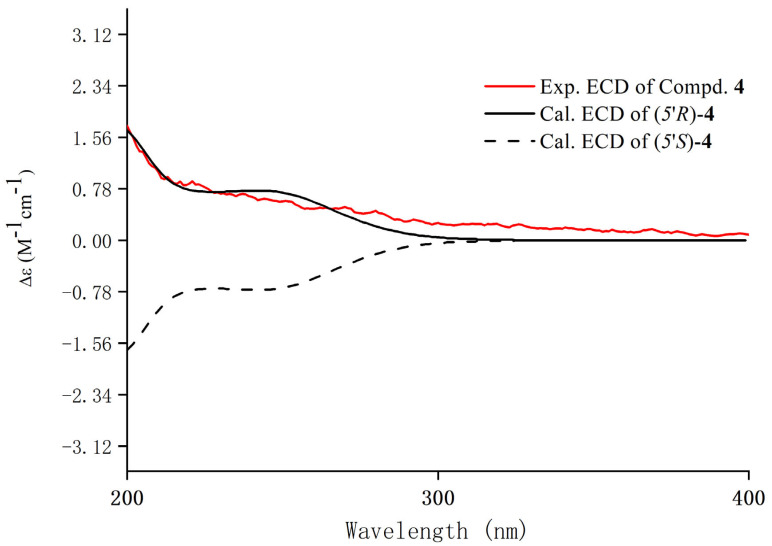
ECD spectra of compound **4**.

Compound **4** was obtained as a yellow-green amorphous powder with ${\left[\alpha \right]}_{D}^{23}$ − 23.4 (*c* 0.10, MeOH) (Figure S35). Its molecular formula, C_12_H_15_NO_4_, was deduced from HRESI-MS at *m*/*z* 236.0926 [M − H]^−^ (calculated for 236.0928) (Figure S33), demonstrating six degrees of unsaturation. The existence of an *N*-ethyl-2-pyrrolidinone group was established by the characteristic carbon signals [*δ*_C_ 177.5 (C-2′), 54.1 (C-5′), 36.3 (C-6′), 32.6 (C-3′), 24.4 (C-4′), 12.6 (C-7′)] observed in the ^13^C NMR spectrum of **4** (Table S4), combined with a comparison of the NMR data with those of **5** [24]. The presence of one trihydroxyphenyl group was deduced from the molecular formula and 1D NMR spectra of **4**. Its *meta*-substitution pattern was indicated by the two aromatic proton signals (*δ*_H_ 5.83, 2H, s, H-3,5). The HMBC correlation from H-5′ (*δ*_H_ 5.36) of the *N*-ethyl-2-pyrrolidinone group to C-1 (*δ*_C_ 105.3) of the phenyl ring confirmed their connection at this position. Thus, the planar structure of **4** was established as 1-ethyl-5-(2,4,6-trihydroxyphenyl)pyrrolidin-2-one, which was further confirmed by the additional 2D NMR correlations detailed in Figure 2. Furthermore, the absolute configuration at C-5′ (*R*) was confirmed by matching the experimental and calculated electronic circular dichroism (ECD) spectra (Figure 4). Therefore, compound **4** was identified as 1-ethyl-(5*R*)-(2,4,6-trihydroxyphenyl)pyrrolidin-2-one, as shown in Figure 1.

### 3.2. Possible Biosynthetic Pathways of the New Compounds

A degradation product of flavan-3-ols lacking the B-ring, (3*R*)-2,5,7-trihydroxy-chroman-3-yl 3,4,5-trihydroxybenzoate, was isolated from black tea by Tanaka et al. in 2003 [11]. No further structural analogs have been reported since then. Fortunately, our phytochemical investigation on black tea made from *C. taliensis* has once again led to the discovery of this class of degradation products, including **1**–**3**. Notably, compounds **1** and **2** also belong to flavoalkaloids, a class of compounds formed by the combination of theanine’s deamination and cyclization product with the A-ring of flavan-3-ols [12,16]. Based on the literature, the formation mechanisms of compounds **1** and **2** were proposed as shown in Figure S38. A key precursor, (3*R*)-2,5,7-trihydroxychroman-3-yl 3,4,5-trihydroxybenzoate, can be generated either from flavan-3-ols (e.g., EGCG) through oxidation and subsequent redox disproportionation or from polymers (e.g., theacitrins A and C) through thermal degradation in black tea [11,27]. Subsequently, compounds **1** and **2** are likely formed through the combination of the A-ring of this precursor with the deamination and cyclization product of theanine [12]. Moreover, they can also be produced directly from the degradation of the components comprising the *N*-ethyl-2-pyrrolidone moiety, for instance, polymers or compound **5**. Compounds **3** and **4** are also hypothesized as degradation products of flavan-3-ols. Additionally, compound **4** may also originate during the processing of black tea produced from *C. taliensis* through the combination of theanine with phloroglucinol, possessing a similar nucleophilic structure to the A-ring of flavan-3-ols.

### 3.3. α-Glucosidase Inhibitory Activity

The *α*-glucosidase inhibitory potential of 15 flavan-3-ols and their derivatives (**1**–**15**) and phloroglucinol was assessed. At 50 µM, all but three compounds (**3**, **4**, and phloroglucinol) displayed significant inhibitory effects on *α*-glucosidase, with inhibition rates exceeding 50%. The IC_50_ values of these active compounds were subsequently determined and detailed in Table 1. Among them, 10 compounds showed superior inhibitory potency to quercetin and acarbose (IC_50_ = 5.75 and 223.3 µM, respectively), with IC_50_ values ranging from 0.09 to 3.57 µM, except for **1**, **2** and **8**, which were less potent than quercetin but still superior to acarbose. The activity order was **15** > **12** > **14** > **13** > **9** > **11** > **10** > **7** > **5** > **6** > quercetin > **1** > **2** > **8** > acarbose. The minimal contribution of the *N*-ethyl-2-pyrrolidinone group to the inhibitory activity against *α*-glucosidase was suggested by the similar activity of **5** and **11**, the weak activity of **4**, and the lack of activity of phloroglucinol. In contrast, the B-ring appears to be a critical structural feature for the *α*-glucosidase inhibitory activity of flavan-3-ols and their derivatives, as indicated by the significantly lower activity of compounds **1** and **2** compared to **5**. The galloyl group at C-3 also appears to be a positive modulator of the activity, as suggested by the significantly lower activity of **8** compared to **10**, which is consistent with previously reported structure–activity relationships for *α*-glucosidase inhibitory activity of flavan-3-ols and their derivatives from black tea [15,19]. Notably, compound **15** exhibited exceptional potency, with an IC_50_ value approximately 60-fold lower than that of quercetin and superior to those of its analogs **13** and **14**. This result may be attributed to its additional galloyl group, which has been proposed to promote favorable π–π T-shaped hydrophobic interactions between the benzene ring of the galloyl group and the amino acid residue Trp406 in the active site of α-glucosidase, thereby increasing the stability of the enzyme–inhibitor complex [28]. Furthermore, existing evidence has suggested that compound **15** may promote lipolysis and *β*-oxidation, and mitigate oxidative stress and inflammatory responses, by activating AMP-activated protein kinase (AMPK), thereby achieving multi-targeted comprehensive regulation of glycolipid metabolism [29].

## 4. Conclusions

In conclusion, a total of 15 flavan-3-ols and their derivatives, including 11 known ones and four new degradation products, were separated and characterized from black tea produced from *C. taliensis*. Among them, compounds **5**, **9** and **12**–**15** were isolated from *C. taliensis* for the first time. The majority of these isolates showed pronounced *α*-glucosidase inhibitory activities. The results revealed that the B-ring of flavan-3-ols is proposed to be a key moiety responsible for *α*-glucosidase inhibition, with the galloyl group at C-3 potentially playing a positive role. In contrast, the *N*-ethyl-2-pyrrolidinone group contributes minimally to this activity. Several possible formation pathways of new flavoalkaloids **1** and **2** have been further hypothesized based on literature research. However, these proposed mechanisms remain subject to experimental confirmation. In short, the further phytochemical study of black tea prepared from *C. taliensis* revealed novel degradation products and other compounds with significant *α*-glucosidase inhibitory activity, supporting the potential of black tea prepared from *C. taliensis* in managing postprandial hyperglycemia. Furthermore, the discovery of degradation products has provided further support for the occurrence of degradation reactions during the processing of black tea, in addition to the well-known oxidation and polymerization reactions.

## Figures and Tables

**Table 1:** The inhibitory activities of **1**–**15** on *α*-glucosidase in black tea produced from *C. taliensis* ^a^.

Sample	IC_50_ (μM) ^b^	Inhibition Ratio (%) ^c^	Sample	IC_50_ (μM) ^b^
quercetin ^d^	5.747 ± 0.797	64.81 ± 3.30 ^e^	**7**	1.679 ± 0.045
acarbose ^d^	223.297 ± 9.983	65.02 ± 1.19 ^f^	**8**	43.403 ± 2.667
phloroglucinol	-	2.69 ± 1.76	**9**	0.418 ± 0.017
**1**	35.879 ± 3.016		**10**	1.430 ± 0.148
**2**	36.566 ± 2.975		**11**	0.647 ± 0.019
**3**	-	31.57 ± 1.67	**12**	0.142 ± 0.003
**4**	-	14.12 ± 0.93	**13**	0.269 ± 0.004
**5**	2.863 ± 0.129		**14**	0.163 ± 0.002
**6**	3.565 ± 0.202		**15**	0.088 ± 0.003

Note: ^a^ Values represent means ± SD (*n* = 3). ^b^ IC_50_ = half-maximal inhibitory concentration to *α*-glucosidase. ^c^ Inhibition ratio (%) at a concentration of 50 µM. ^d^ Positive control. ^e^ Tested concentration: 10 µM. ^f^ Tested concentration: 400 µM. “-” refers to not detected. All pairwise comparisons of IC_50_ values among the tested compounds were statistically significant (*p* < 0.05, Student’s *t*-test).

## Data Availability

The original contributions presented in the study are included in the article/Supplementary Material, further inquiries can be directed to the corresponding authors.

## Supplementary Materials

The following supporting information can be downloaded at: https://www.mdpi.com/article/10.3390/foods15091609/s1, Figures S1–S6: NMR spectrum of compound **1** in CD_3_OD; Figure S7: HRESI-MS spectrum of compound **1**; Figure S8: CD and UV spectra of compound **1** in MeOH; Figure S9: OR of compound **1** in MeOH; Figures S10–S14: NMR spectrum of compound **2** in CD_3_OD; Figure S15: HRESI-MS spectrum of compound **2**; Figure S16: CD and UV spectra of compound **2** in MeOH; Figure S17: OR of compound **2** in MeOH; Figures S18–S23: NMR spectrum of compound **3** in CD_3_OD; Figure S24: HRESI-MS spectrum of compound **3**; Figure S25: CD and UV spectra of compound **3** in MeOH; Figure S26: OR of compound **3** in MeOH; Figures S27–S32: NMR spectrum of compound **4** in CD_3_OD; Figure S33: HRESI-MS spectrum of compound **4**; Figure S34: CD and UV spectra of compound **4** in MeOH; Figure S35: OR of compound **4** in MeOH; Figures S36 and S37: NMR spectrum of compound **6** in CD_3_OD; Figure S38: Possible formation mechanisms of compounds **1** and **2**; Table S1: ^13^C and ^1^H NMR spectroscopic data of compound **1** in CD_3_OD; Table S2: ^13^C and ^1^H NMR spectroscopic data of compound **2** in CD_3_OD; Table S3: ^13^C and ^1^H NMR spectroscopic data of compounds **3** and **6** in CD_3_OD; Table S4: ^13^C and ^1^H NMR spectroscopic data of compound **4** in CD_3_OD.
