# Supplementary material for: Four New Flavan-3-ol Derivatives with Potent α-Glucosidase Inhibitory Activity from Black Tea Produced from Camellia taliensis
Source: Foods. 2026 May 6;15(9):1609. doi: 10.3390/foods15091609 (PMC13163379; doi:10.3390/foods15091609)
Supplement: Supplementary file 1 [file foods-15-01609-s001.zip › foods-4258431-supplementary.pdf]

Supplementary data contents page

Four New Flavan-3-ol Derivatives with Potent *α*-Glucosidase Inhibitory Activity from Black Tea Produced from *Camellia taliensis*

Min Chen [1,2,†], Na Li [3,†,*], Jia-Huan Shang [1], Hong-Tao Zhu [3], Zhao-Hong Duan [4] and Ying-Jun Zhang [1,*]

[1] *State Key Laboratory of Phytochemistry & Natural Medicines, Kunming Institute of Botany, Chinese Academy of Sciences, Kunming 650201, People's Republic of China.*

[2] *University of Chinese Academy of Sciences, Beijing 100049, People's Republic of China.*

[3] *Key Laboratory of Phytochemistry and Plant Resources in West China, Kunming Institute of Botany, Chinese Academy of Sciences, Kunming 650201, People's Republic of China.*

[4] *Tea Training Department, Zhao-Hong Gu-Shu Training Center, Lincang 675911, People's Republic of China*

1. Figure S1. ^{1}H NMR spectrum of compound **1** in CD_3OD
2. Figure S2. ^{13}C NMR spectrum of compound **1** in CD_3OD
3. Figure S3. HSQC spectrum of compound **1** in CD_3OD
4. Figure S4. HMBC spectrum of compound **1** in CD_3OD
5. Figure S5. COSY spectrum of compound **1** in CD_3OD
6. Figure S6. ROESY spectrum of compound **1** in CD_3OD
7. Figure S7. HRESI-MS spectrum of compound **1**
8. Figure S8. CD and UV spectra of compound **1** in MeOH
9. Figure S9. OR of compound **1** in MeOH
10. Figure S10. ^{1}H NMR spectrum of compound **2** in CD_3OD
11. Figure S11. ^{13}C NMR spectrum of compound **2** in CD_3OD
12. Figure S12. HSQC spectrum of compound **2** in CD_3OD
13. Figure S13. HMBC spectrum of compound **2** in CD_3OD
14. Figure S14. COSY spectrum of compound **2** in CD_3OD
15. Figure S15. HRESI-MS spectrum of compound **2**
16. Figure S16. CD and UV spectra of compound **2** in MeOH
17. Figure S17. OR of compound **2** in MeOH
18. Figure S18. ^{1}H NMR spectrum of compound **3** in CD_3OD

19. Figure S19. ^{13}C NMR spectrum of compound **3** in CD_3OD
20. Figure S20. HSQC spectrum of compound **3** in CD_3OD
21. Figure S21. HMBC spectrum of compound **3** in CD_3OD
22. Figure S22. COSY spectrum of compound **3** in CD_3OD
23. Figure S23. ROESY spectrum of compound **3** in CD_3OD
24. Figure S24. HRESI-MS spectrum of compound **3**
25. Figure S25. CD and UV spectra of compound **3** in MeOH
26. Figure S26. OR of compound **3** in MeOH
27. Figure S27. ^{1}H NMR spectrum of compound **4** in CD_3OD
28. Figure S28. ^{13}C NMR spectrum of compound **4** in CD_3OD
29. Figure S29. HSQC spectrum of compound **4** in CD_3OD
30. Figure S30. HMBC spectrum of compound **4** in CD_3OD
31. Figure S31. COSY spectrum of compound **4** in CD_3OD
32. Figure S32. ROESY spectrum of compound **4** in CD_3OD
33. Figure S33. HRESI-MS spectrum of compound **4**
34. Figure S34. CD and UV spectra of compound **4** in MeOH
35. Figure S35. OR of compound **4** in MeOH
36. Figure S36. ^{1}H NMR spectrum of compound **6** in CD_3OD
37. Figure S37. ^{13}C NMR spectrum of compound **6** in CD_3OD
38. Figure S38. Possible formation mechanisms of compounds **1** and **2**
39. Table S1. ^{13}C and ^{1}H NMR spectroscopic data of compound **1** in CD_3OD
40. Table S2. ^{13}C and ^{1}H NMR spectroscopic data of compound **2** in CD_3OD
41. Table S3. ^{13}C and ^{1}H NMR spectroscopic data of compounds **3** and **6** in CD_3OD
42. Table S4. ^{13}C and ^{1}H NMR spectroscopic data of compound **4** in CD_3OD

Figure S1. ^{1}H NMR spectrum of compound **1** in CD_3OD

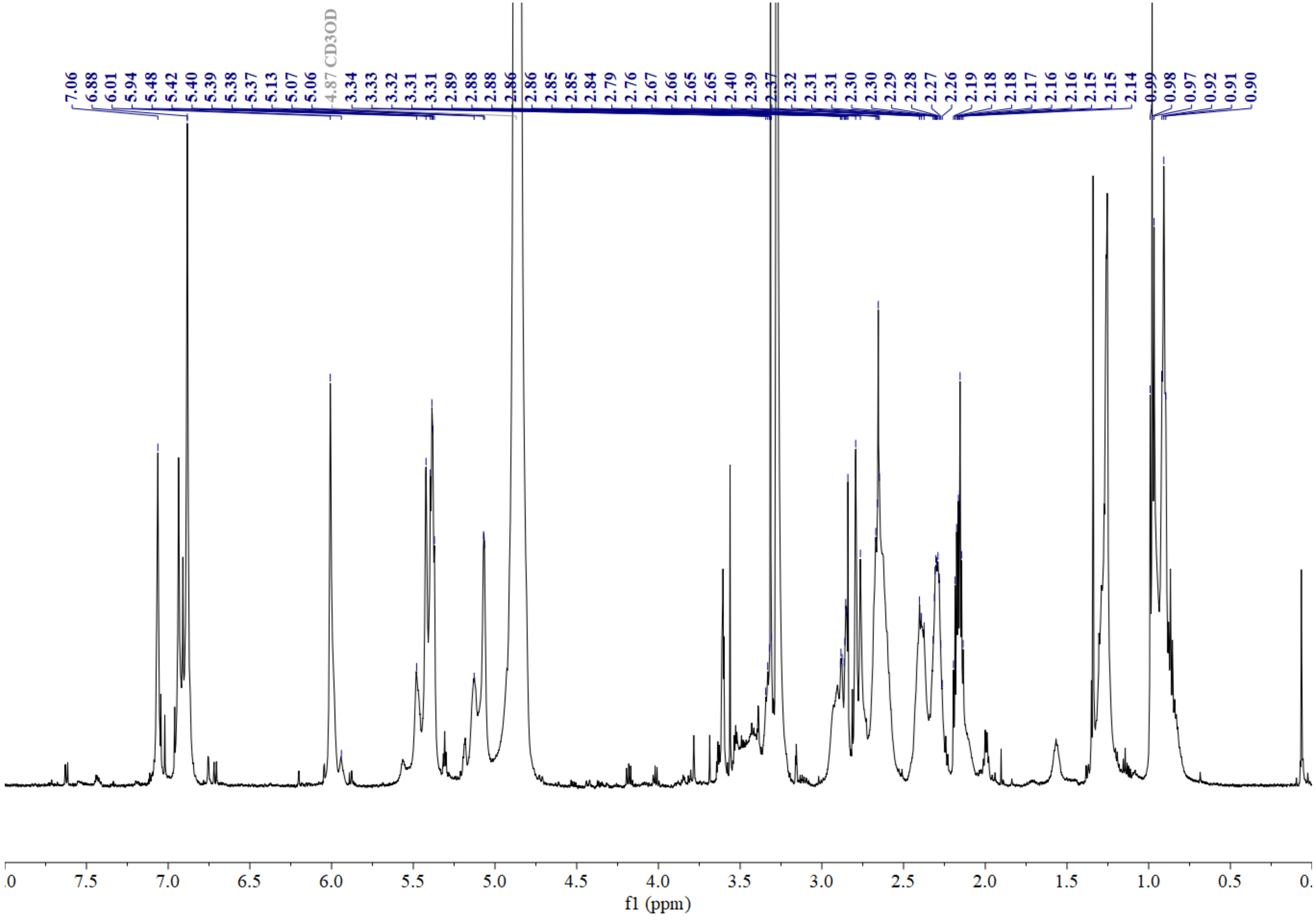

Figure S2. ^{13}C NMR spectrum of compound **1** in CD_3OD

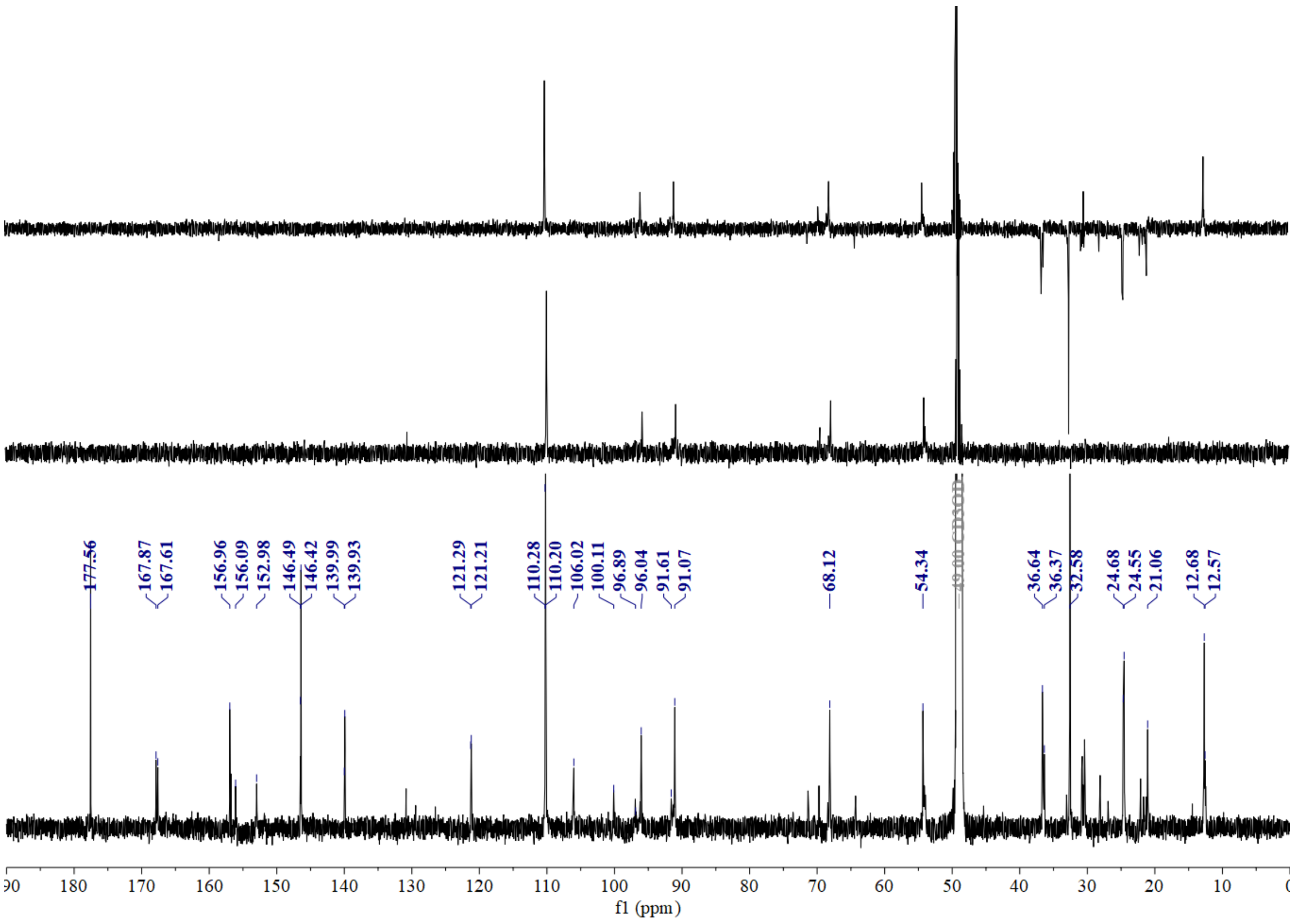

Figure S3. HSQC spectrum of compound **1** in CD_3OD

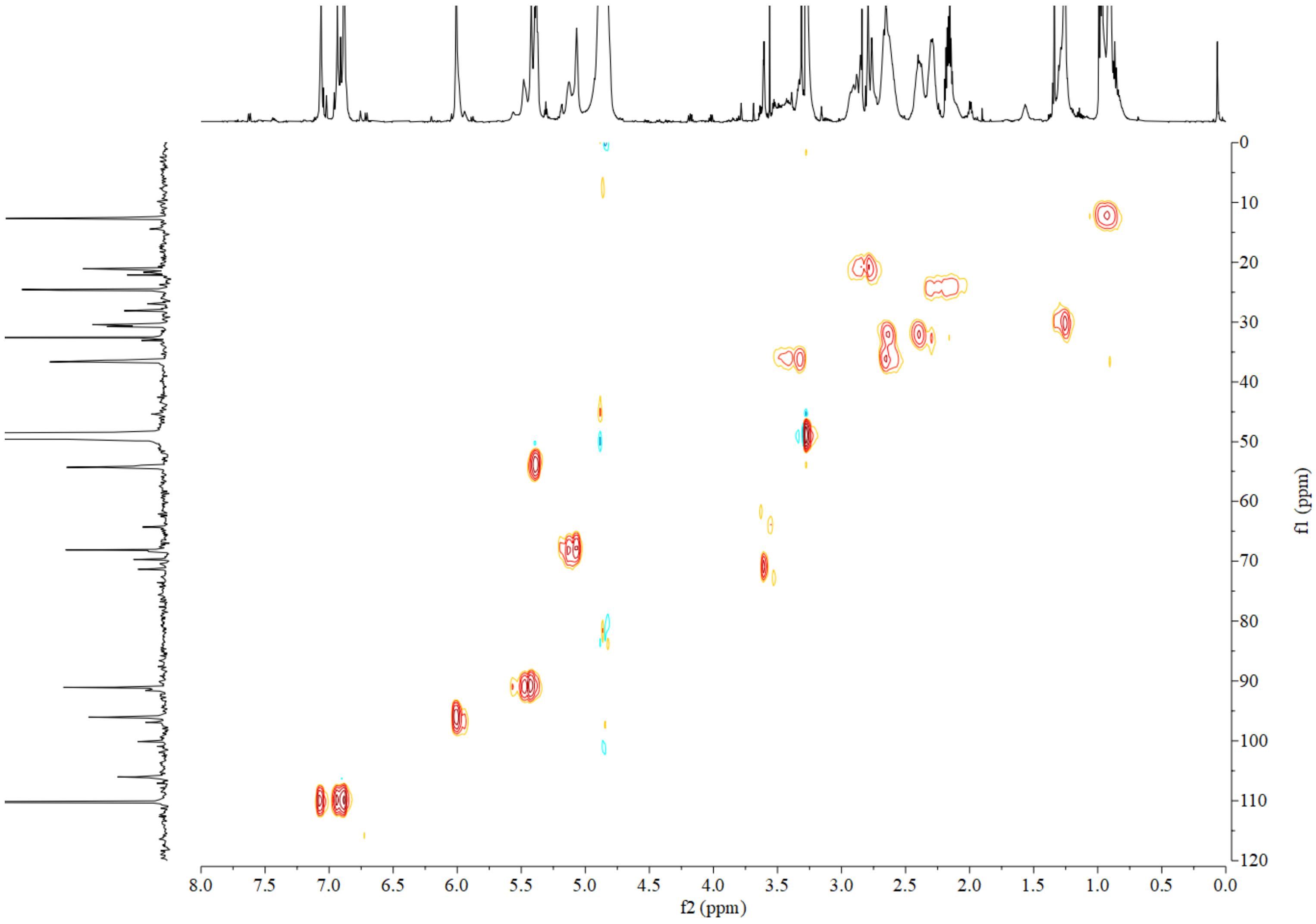

Figure S4. HMBC spectrum of compound **1** in CD_3OD

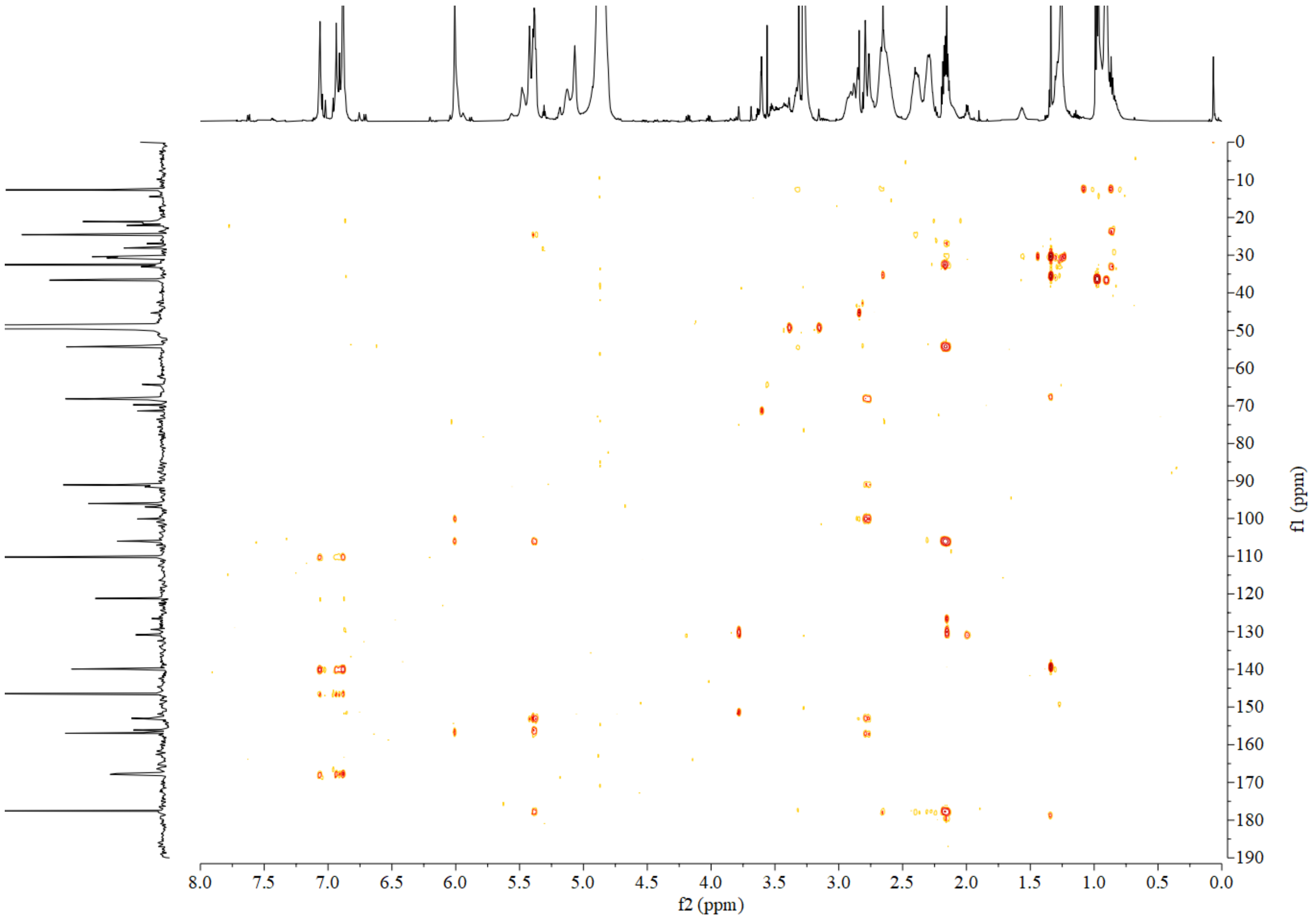

Figure S5. COSY spectrum of compound **1** in CD_3OD

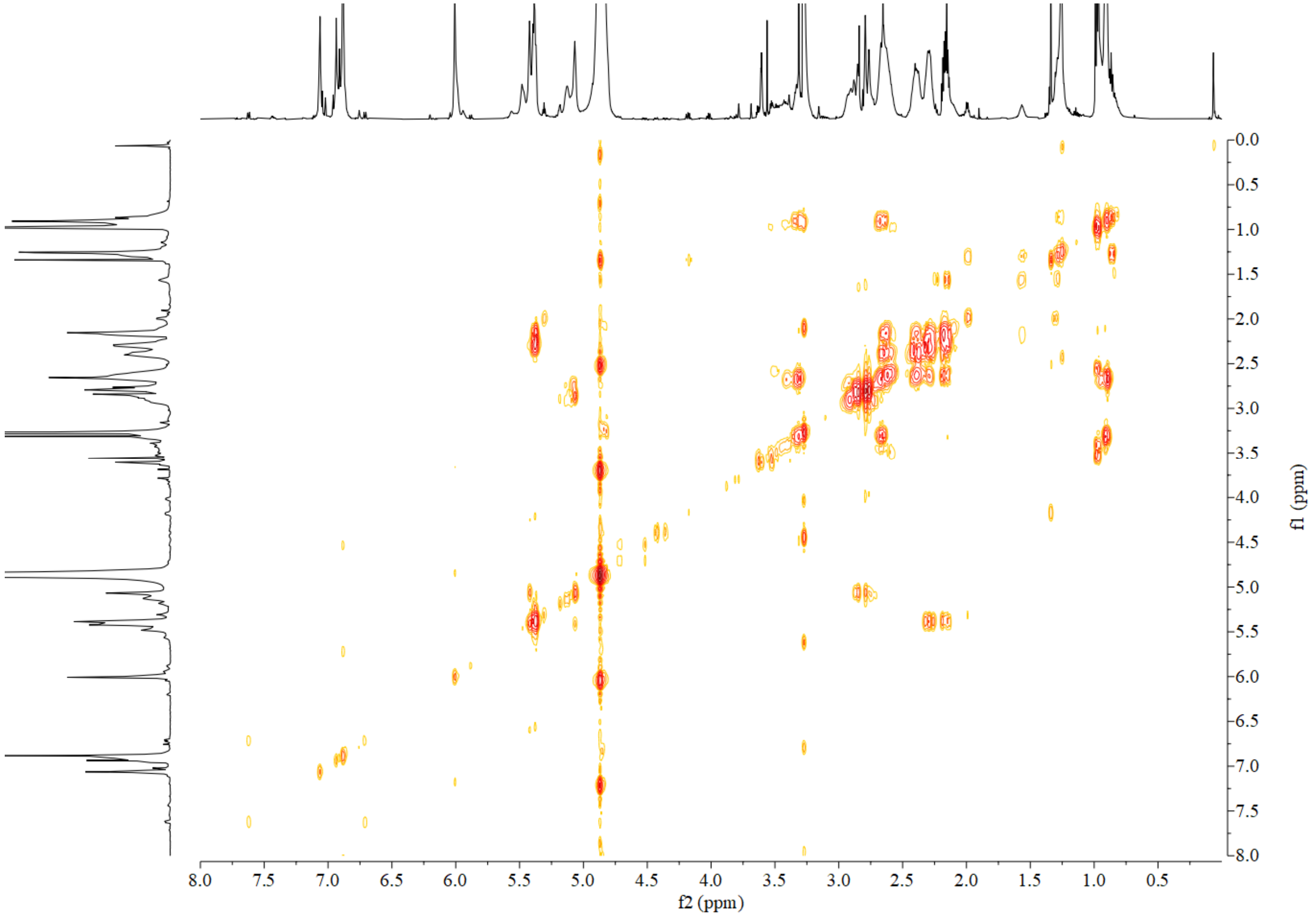

Figure S6. ROESY spectrum of compound **1** in CD_3OD

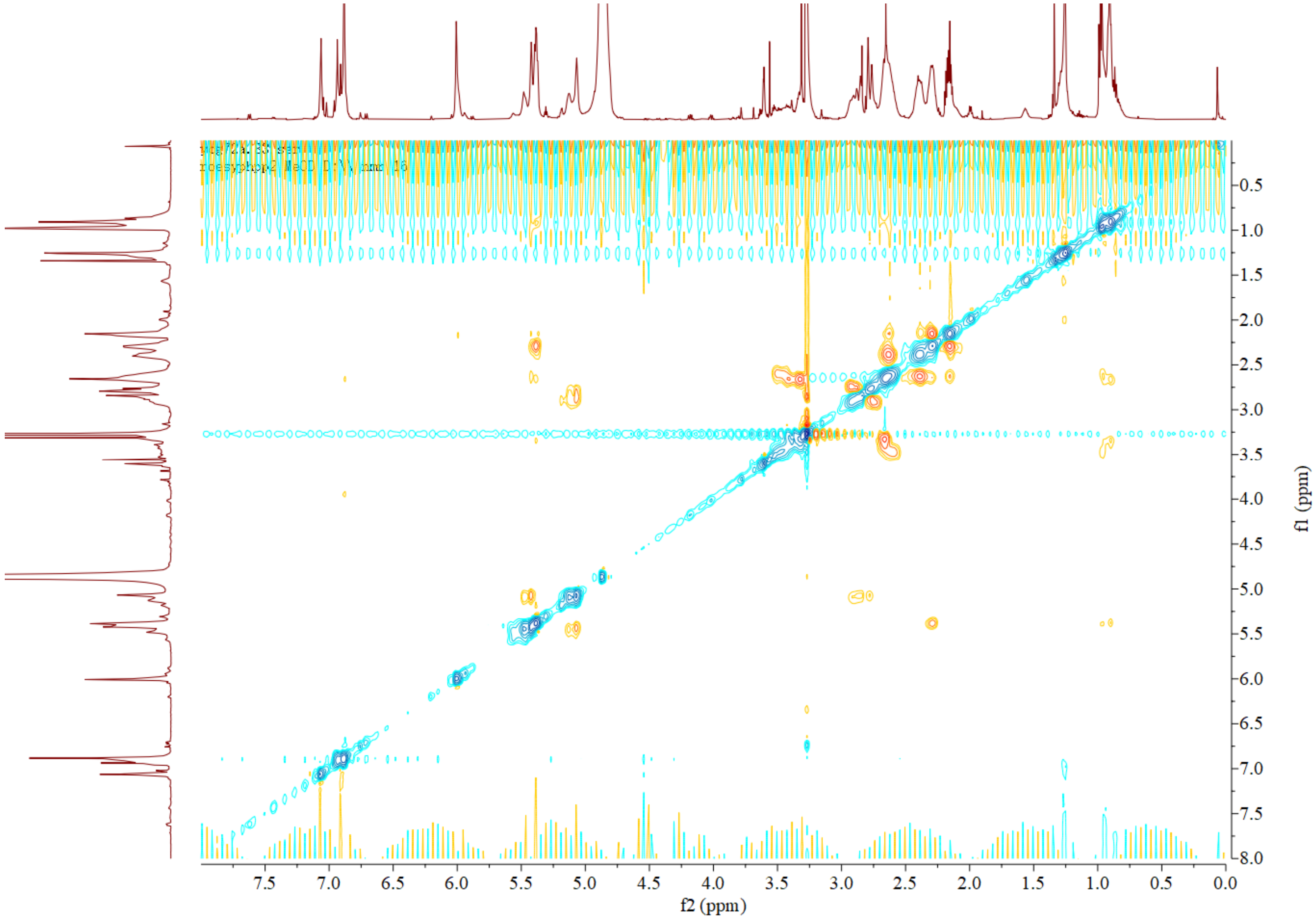

Figure S7. HRESI-MS spectrum of compound **1**

Formula Predictor Report - ycg72a.lcd

Data File: E:\DATA\2023\0112\ycg72a.lcd

Elmt	Val.	Min	Max	Elmt	Val.	Min	Max	Elmt	Val.	Min	Max	Elmt	Val.	Min	Max	Use Adduct
H	1	10	100	F	1	0	0	Cl	1	0	0	Ag	1	0	0	H
2H	1	0	0	Na	1	0	0	Co	2	0	0	I	3	0	0	Cl
B	3	0	0	Mg	2	0	0	Cu	2	0	0	Ir	3	0	0	
C	4	5	50	Si	4	0	0	Se	2	0	0					
N	3	0	10	P	3	0	0	Br	1	0	0					
O	2	0	30	S	2	0	0	Pd	2	0	0					

Error Margin (ppm):	5	DBE Range:	not fixed	Electron Ions:	both
HC Ratio:	unlimited	Apply N Rule:	no	Use MSn Info:	yes
Max Isotopes:	all	Isotope RI (%):	1.00	Isotope Res:	10000
MSn Iso RI (%):	75.00	MSn Logic Mode:	OR	Max Results:	30

Event#: 2 MS(E-) Ret. Time : 0.440 -> 0.547 Scan# : 68 -> 84

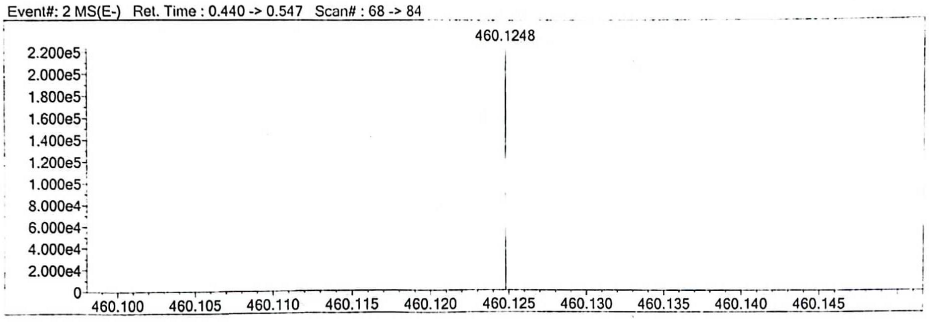

Measured region for 460.1248 m/z

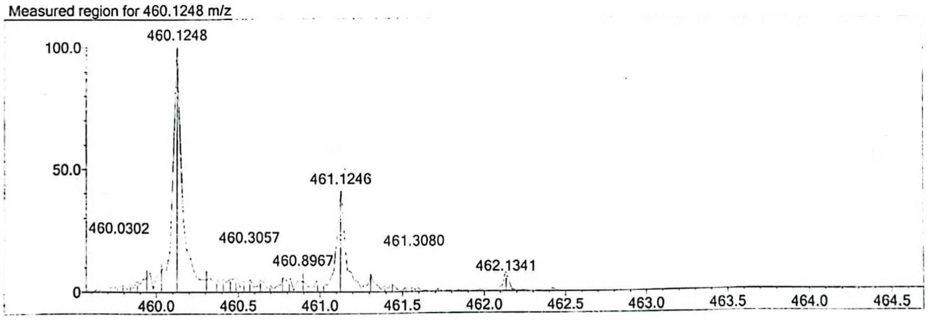

C22 H23 N O10 [M-H]- : Predicted region for 460.1249 m/z

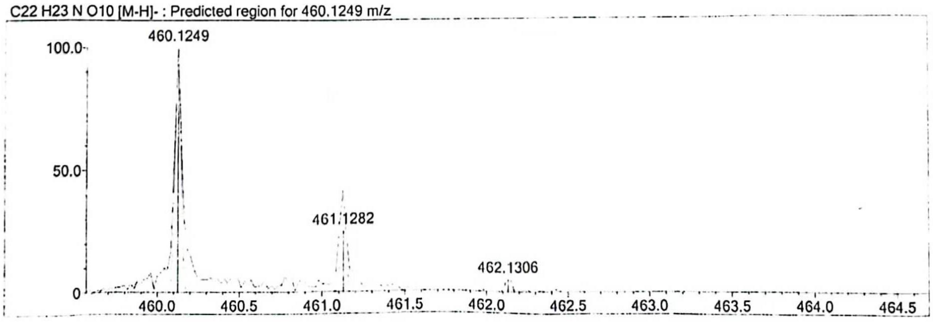

Formula (M)	Ion	Meas. m/z	Pred. m/z	Df. (mDa)	Df. (ppm)	DBE
C22 H23 N O10	[M-H]-	460.1248	460.1249	-0.1	-0.22	12.0

Figure S8. CD and UV spectra of compound **1** in MeOH

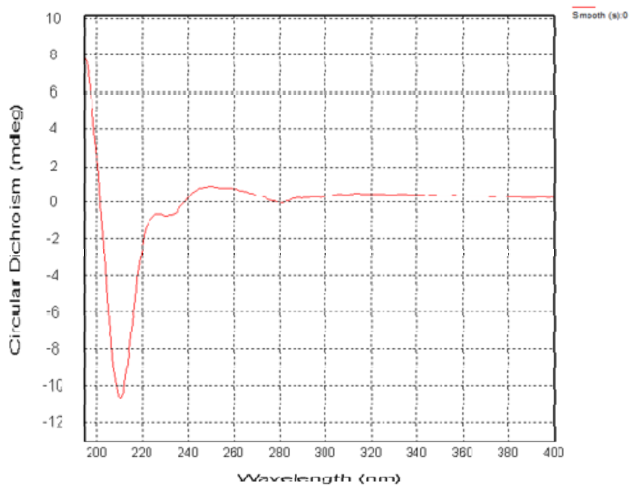

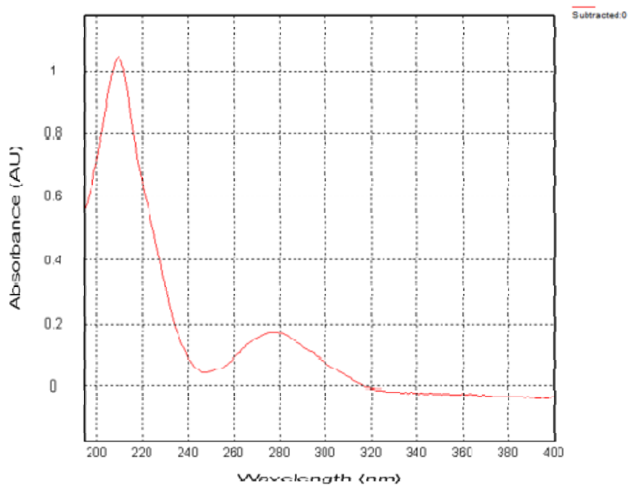

File: YCG72A-1mm(195-400)23011619.dsx
ProBinaryX
Attributes :
- Time Stamp :Mon Jan 16 16:53:12 2023

- File ID : {4E6E6181-A69C-4bb2-90D3-69173AE786F8}
- Is CFR Compliant : false
- Original unaltered data

Remarks:
- User: CD
- Date: 2023/01/16
- Instrument: 0547
- DetectorType: LAAPD
- DichOS Calibration Correction Curve: 0547/2
- HV (CDDC channel): 0 v
- Time per point: 1 s
- Description: Sample 1
- Concentration: 0.1022mg/mLMeOH
- Pathlength: 1 mm
- Temperature: 20°C

Settings:
- HV
- Time-per-point: 1s (25us x 40000)
- SE
- Wavelength: 195nm - 400nm
- Step Size: 1nm
- Bandwidth: 1nm

Figure S9. OR of compound **1** in MeOH

Rudolph Research Analytical

This sample was measured on an Autopol VI, Serial #91058
Manufactured by Rudolph Research Analytical, Hackettstown, NJ, USA.

Measurement Date : Monday, 16-JAN-2023

Set Temperature : 20.0

Time Delay : Disabled

Delay between Measurement : Disabled

n	Average	Std.Dev.	% RSD	Maximum	Minimum
5	-56.02	0.30	-0.53	-55.48	-56.16

S.No	Sample ID	Time	Result	Scale	OR °Arc	WLG.nm	Lg.mm	Conc.g/100ml	Temp.
1	YCG72A	01:39:43 PM	-56.16	SR	-0.082	589	100.00	0.146	20.2
2	YCG72A	01:39:50 PM	-56.16	SR	-0.082	589	100.00	0.146	20.1
3	YCG72A	01:39:57 PM	-56.16	SR	-0.082	589	100.00	0.146	20.1
4	YCG72A	01:40:03 PM	-56.16	SR	-0.082	589	100.00	0.146	20.0
5	YCG72A	01:40:10 PM	-55.48	SR	-0.081	589	100.00	0.146	20.0

Figure S10. ^{1}H NMR spectrum of compound **2** in CD_3OD

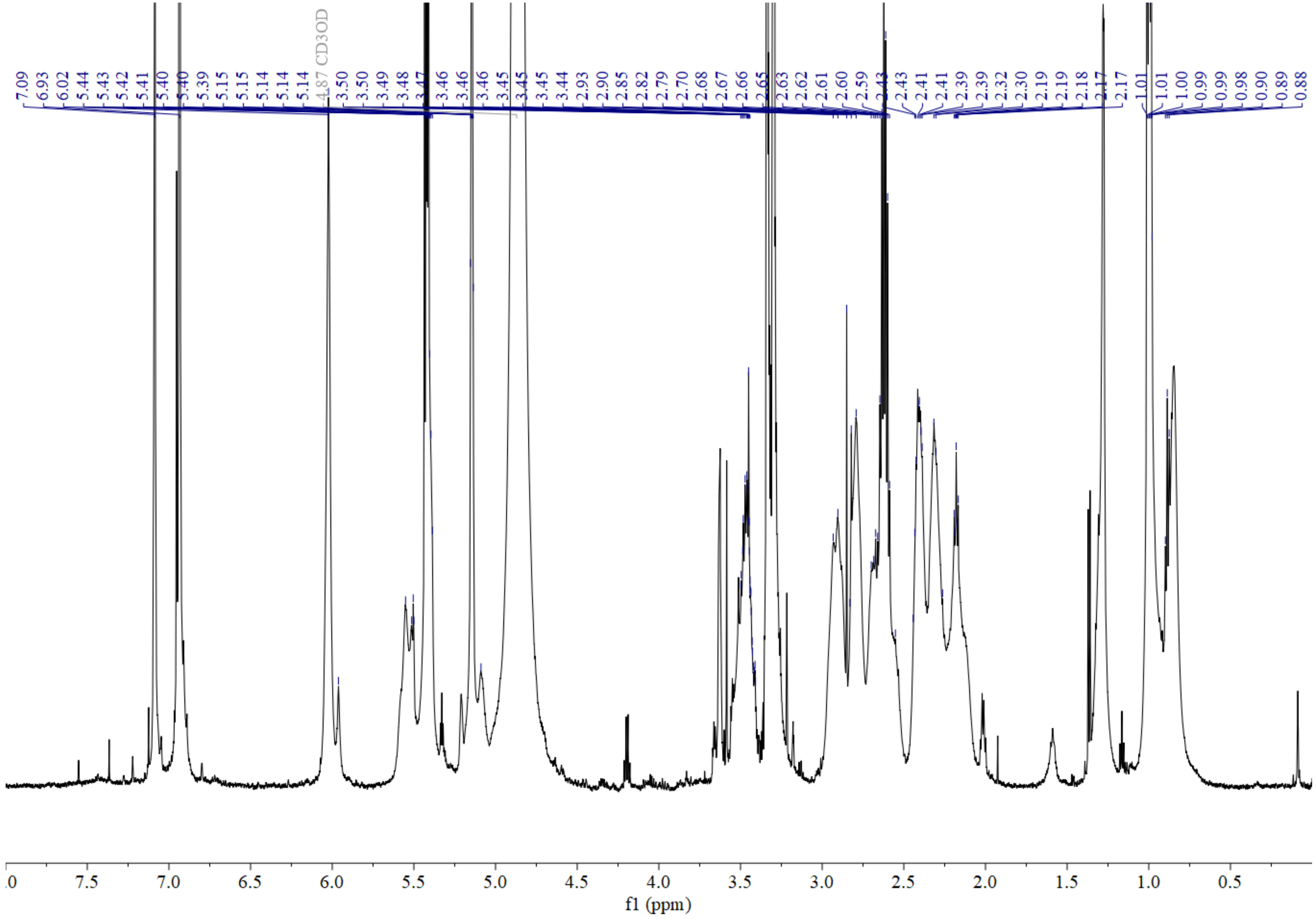

Figure S11. ^{13}C NMR spectrum of compound **2** in CD_3OD

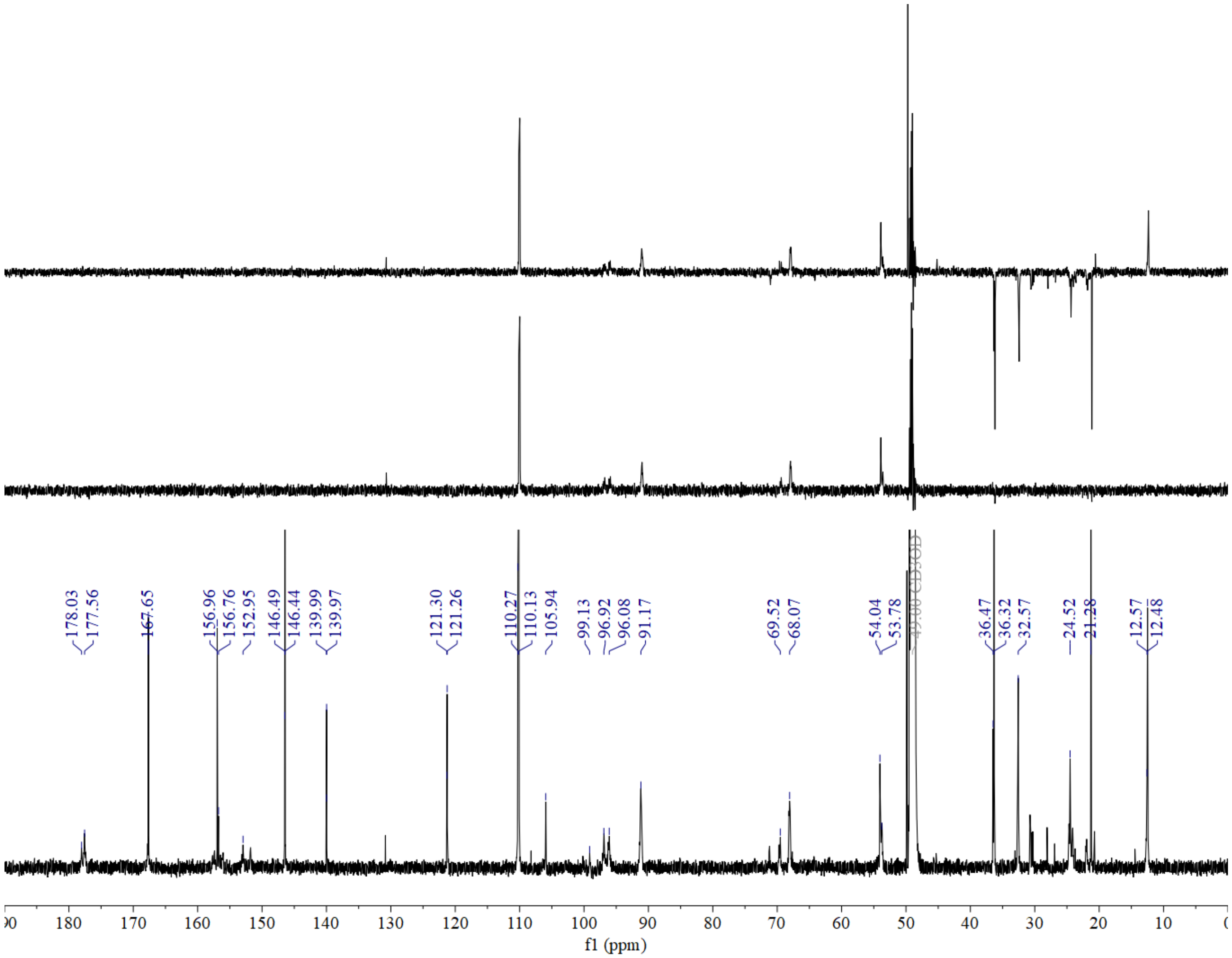

Figure S12. HSQC spectrum of compound **2** in CD_3OD

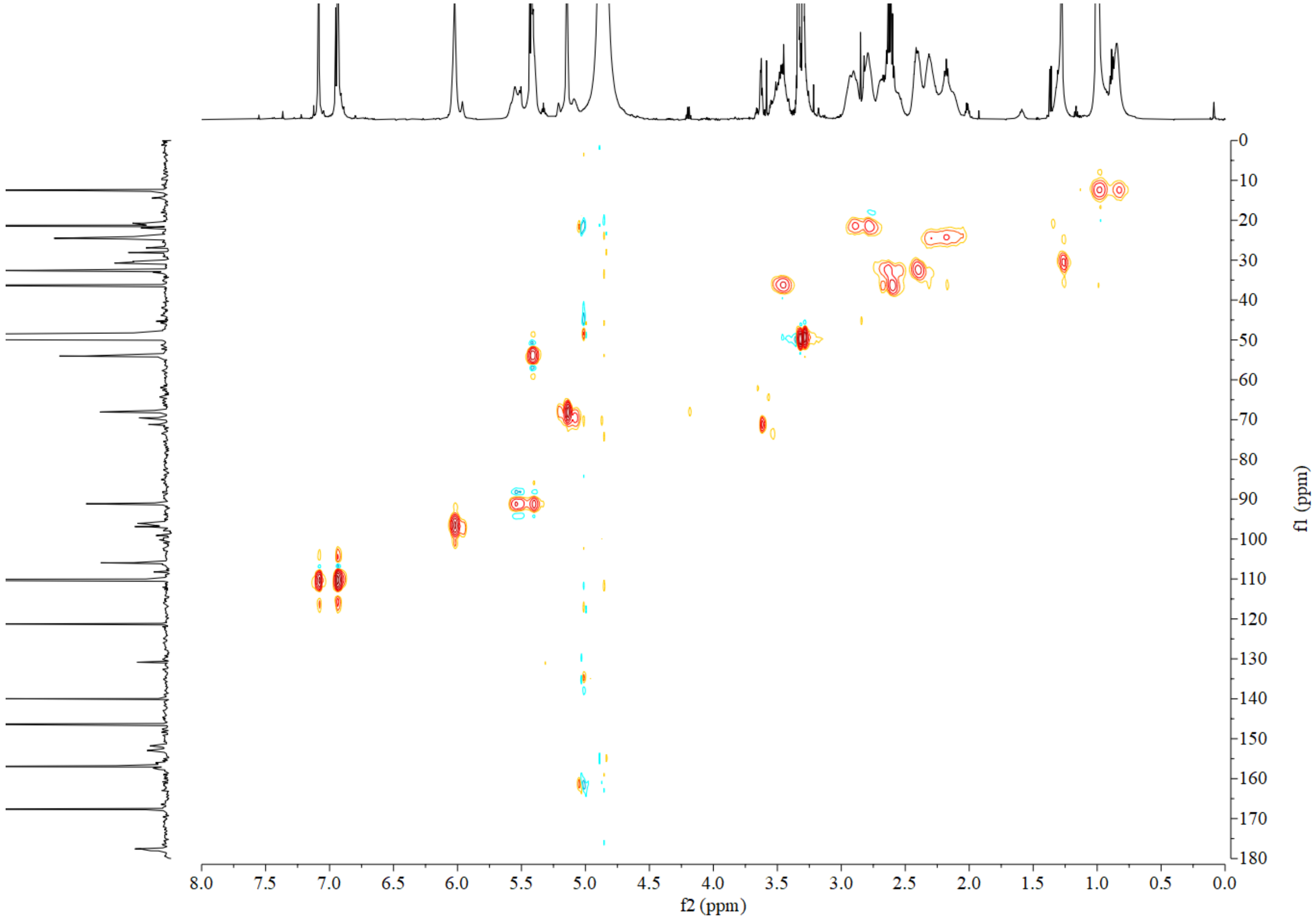

Figure S13. HMBC spectrum of compound **2** in CD_3OD

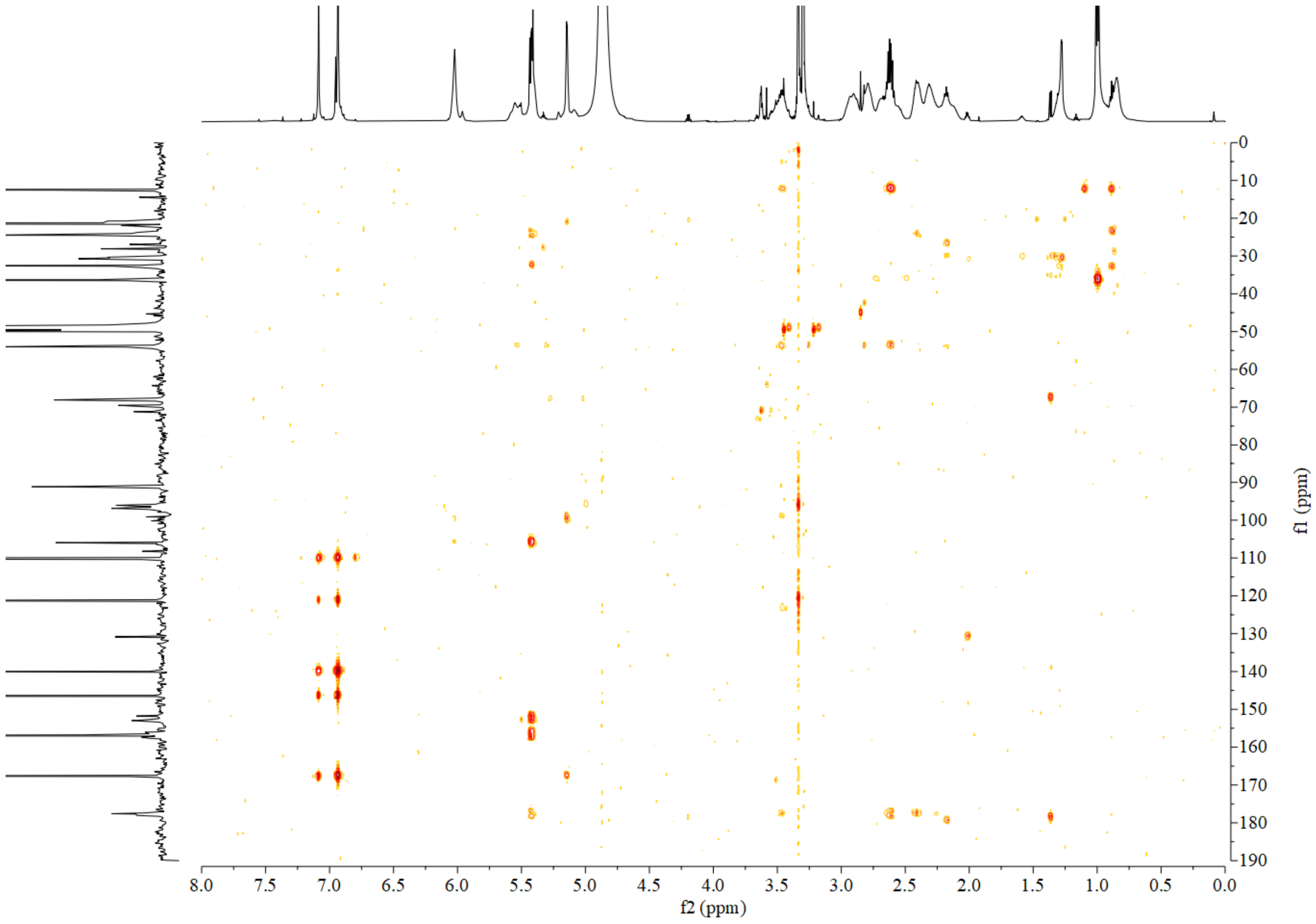

Figure S14. COSY spectrum of compound **2** in CD_3OD

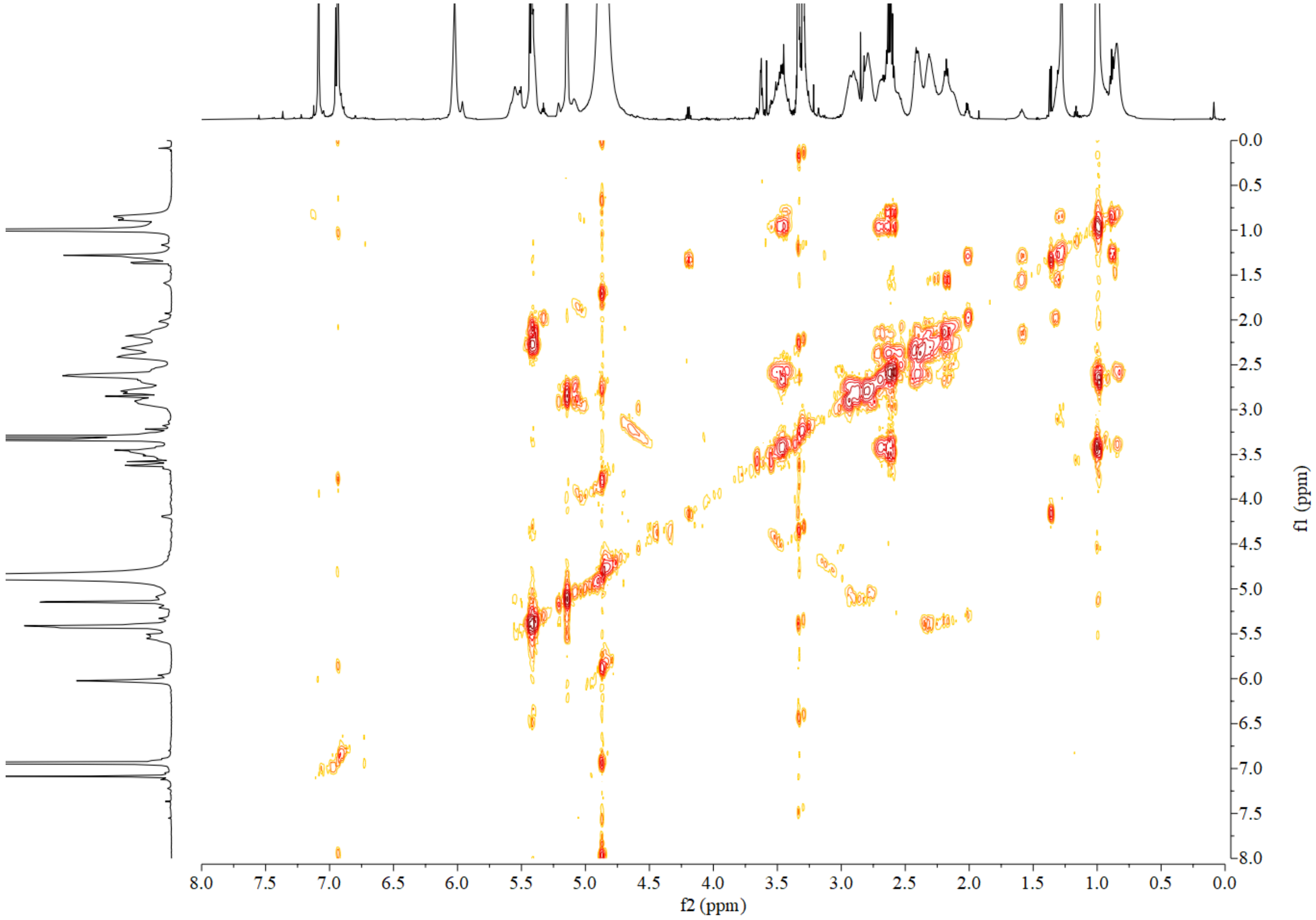

Figure S15. HRESI-MS spectrum of compound **2**

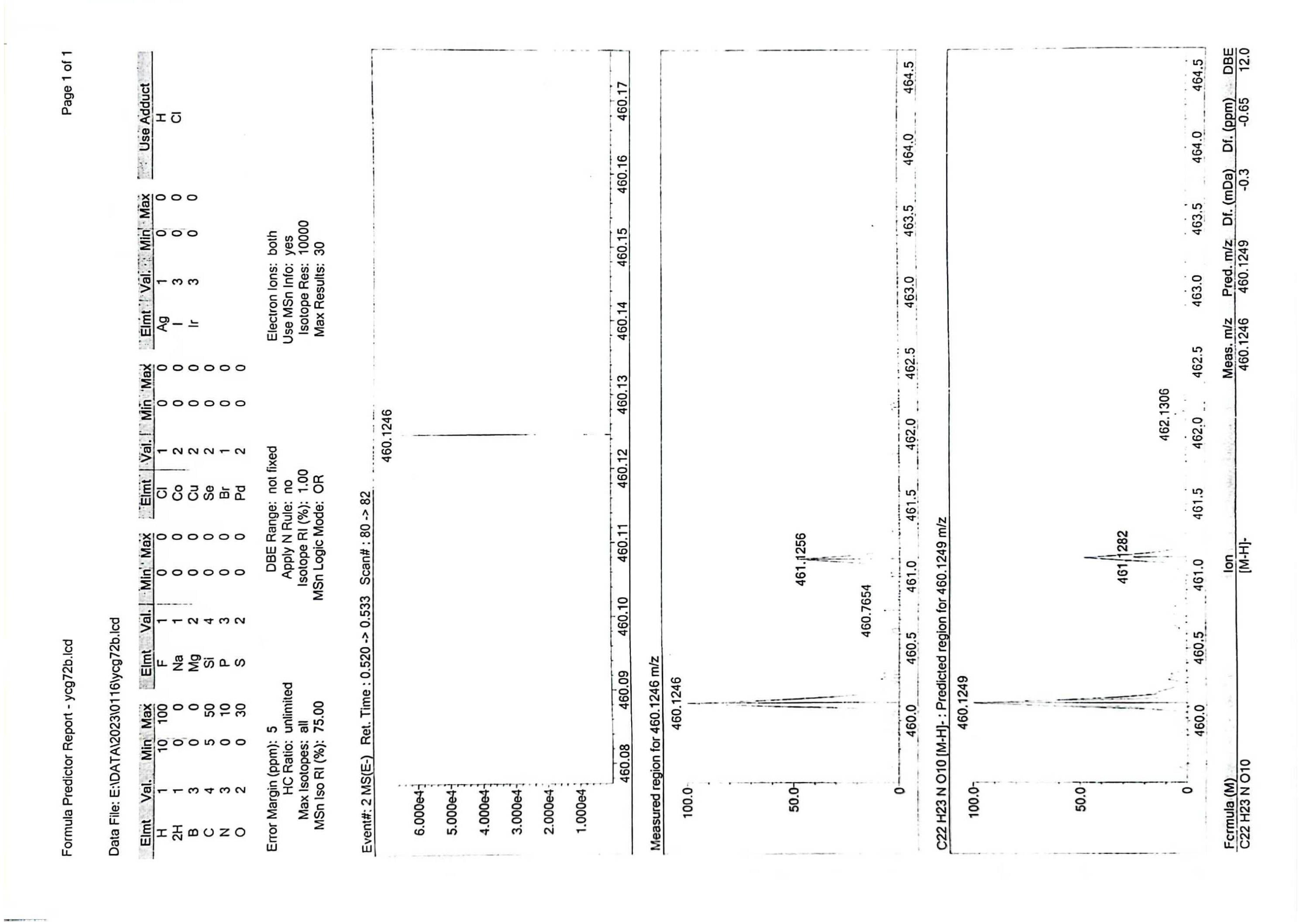

Formula Predictor Report - ycg72b.lcd

Page 1 of 1

Data File: E:\DATA\2023\0116\ycg72b.lcd

Elmt	Val.	Min	Max	Elmt	Val.	Min	Max	Elmt	Val.	Min	Max	Elmt	Val.	Min	Max	Use Adduct
H	1	10	100	F	1	0	0	Cl	1	0	0	Ag	1	0	0	H
2H	1	0	0	Na	1	0	0	Co	2	0	0	I	3	0	0	Cl
B	3	0	0	Mg	2	0	0	Cu	2	0	0	Ir	3	0	0	
C	4	5	50	Si	4	0	0	Se	2	0	0					
N	3	0	10	P	3	0	0	Br	1	0	0					
O	2	0	30	S	2	0	0	Pd	2	0	0					

Error Margin (ppm): 5
HC Ratio: unlimited
Max Isotopes: all
MSn Iso RI (%): 75.00

DBE Range: not fixed
Apply N Rule: no
Isotope RI (%): 1.00
MSn Logic Mode: OR

Electron Ions: both
Use MSn Info: yes
Isotope Res: 10000
Max Results: 30

Formula (M)	Ion	Meas. m/z	Pred. m/z	Df. (mDa)	Df. (ppm)	DBE
C22 H23 N O10	[M-H]-	460.1246	460.1249	-0.3	-0.65	12.0

Figure S16. CD and UV spectra of compound **2** in MeOH

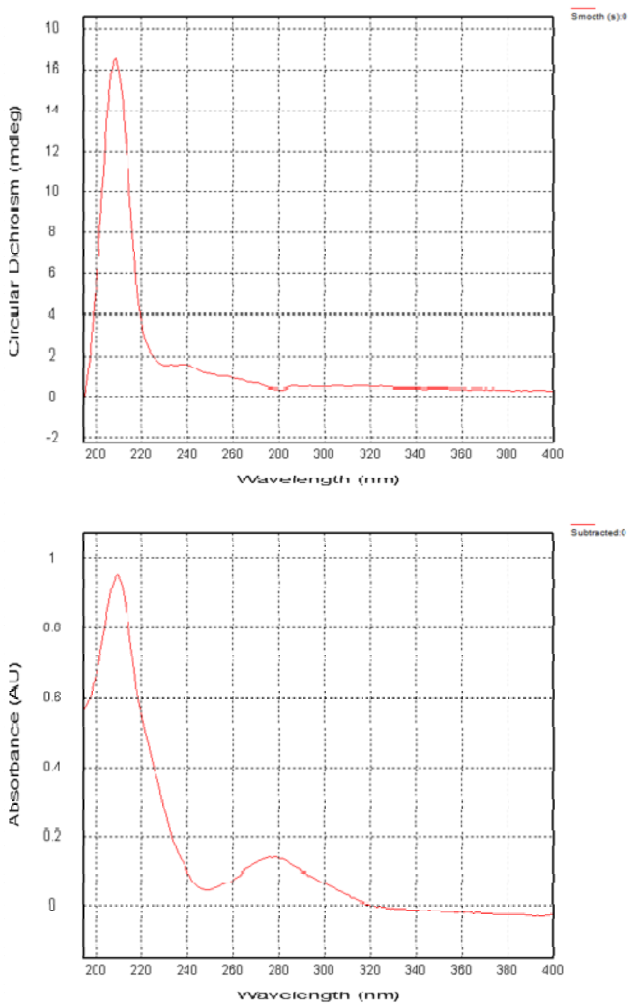

File: YCG72B-1mm(195-600)23011620.dsx
ProBinaryX
Attributes :
- Time Stamp :Mon Jan 16 17:18:05 2023

- File ID : {0F594EC9-15BC-4bc3-A459-99C874BE98DE}
- Is CFR Compliant : false
- Original unaltered data
Remarks:
- User: CD
- Date: 2023/01/16
- Instrument: 0547
- DetectorType: LAAPD
- DichOS Calibration Correction Curve: 0547/2
- HV (CDDC channel): 0 v
- Time per point: 1 s
- Description: Sample 1
- Concentration: 0.0819mg/mL MeOH
- Pathlength: 1 mm
- Temperature: 20°C

Settings:
- HV
- Time-per-point: 1s (25us x 40000)
- SE
- Wavelength: 195nm - 400nm
- Step Size: 1nm
- Bandwidth: 1nm

Figure S17. OR of compound **2** in MeOH

Rudolph Research Analytical

This sample was measured on an Autopol VI, Serial #91058
Manufactured by Rudolph Research Analytical, Hackettstown, NJ, USA.

Measurement Date : Friday, 10-FEB-2023

Set Temperature : OFF

Time Delay : Disabled

Delay between Measurement : Disabled

n	Average	Std.Dev.	% RSD	Maximum	Minimum
5	2.80	1.34	47.85	4.15	1.38

S.No	Sample ID	Time	Result	Scale	OR °Arc	WLG.nm	Lg.mm	Conc.g/100ml	Temp.
1	YCG72B	02:24:58 PM	3.23	SR	0.0021	589	100.00	0.065	19.3
2	YCG72B	02:25:06 PM	1.38	SR	0.0009	589	100.00	0.065	19.3
3	YCG72B	02:25:14 PM	3.85	SR	0.0025	589	100.00	0.065	19.3
4	YCG72B	02:25:23 PM	4.15	SR	0.0027	589	100.00	0.065	19.3
5	YCG72B	02:25:31 PM	1.38	SR	0.0009	589	100.00	0.065	19.3

Figure S18. ^{1}H NMR spectrum of compound **3** in CD_3OD

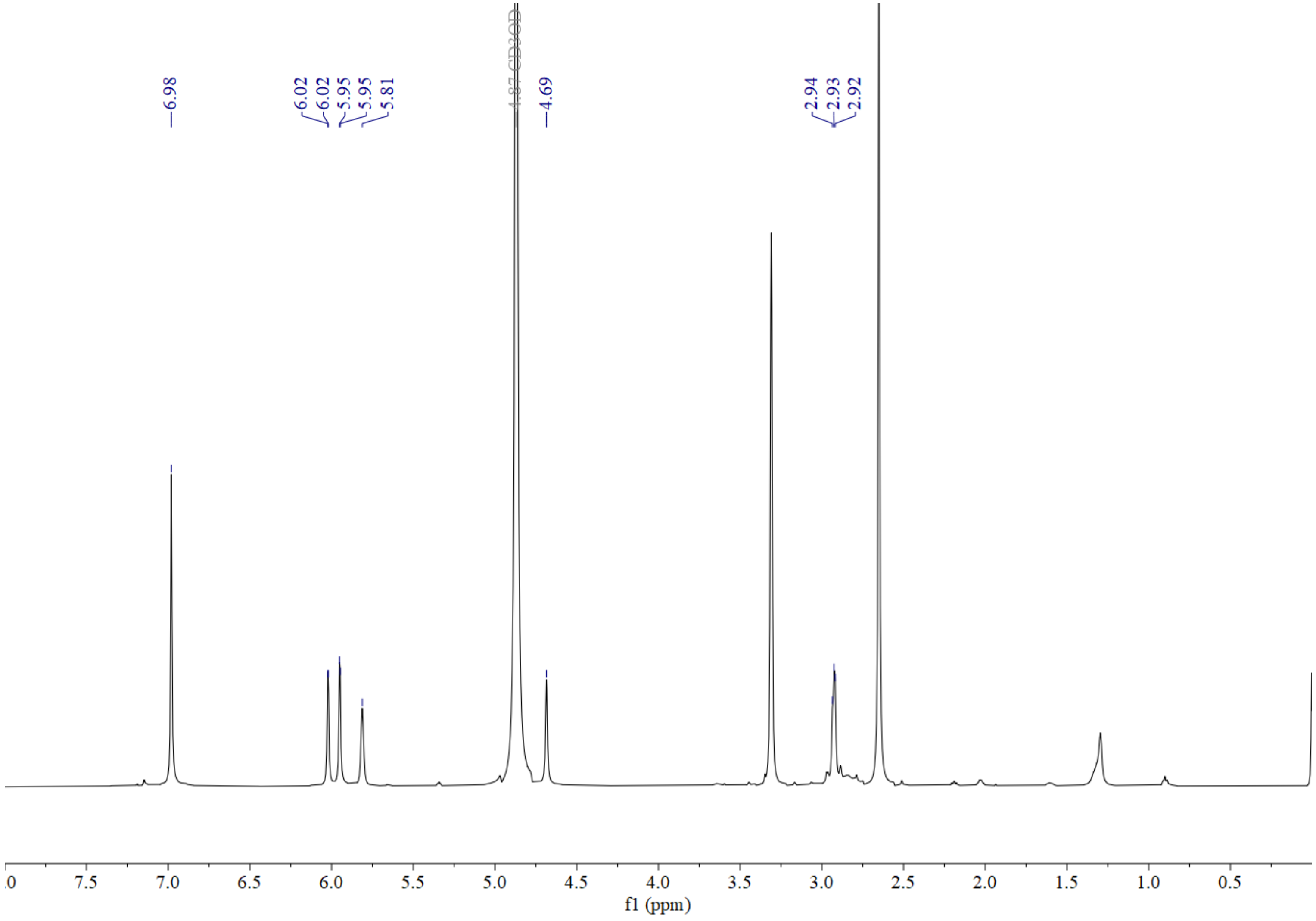

Figure S19. ^{13}C NMR spectrum of compound **3** in CD_3OD

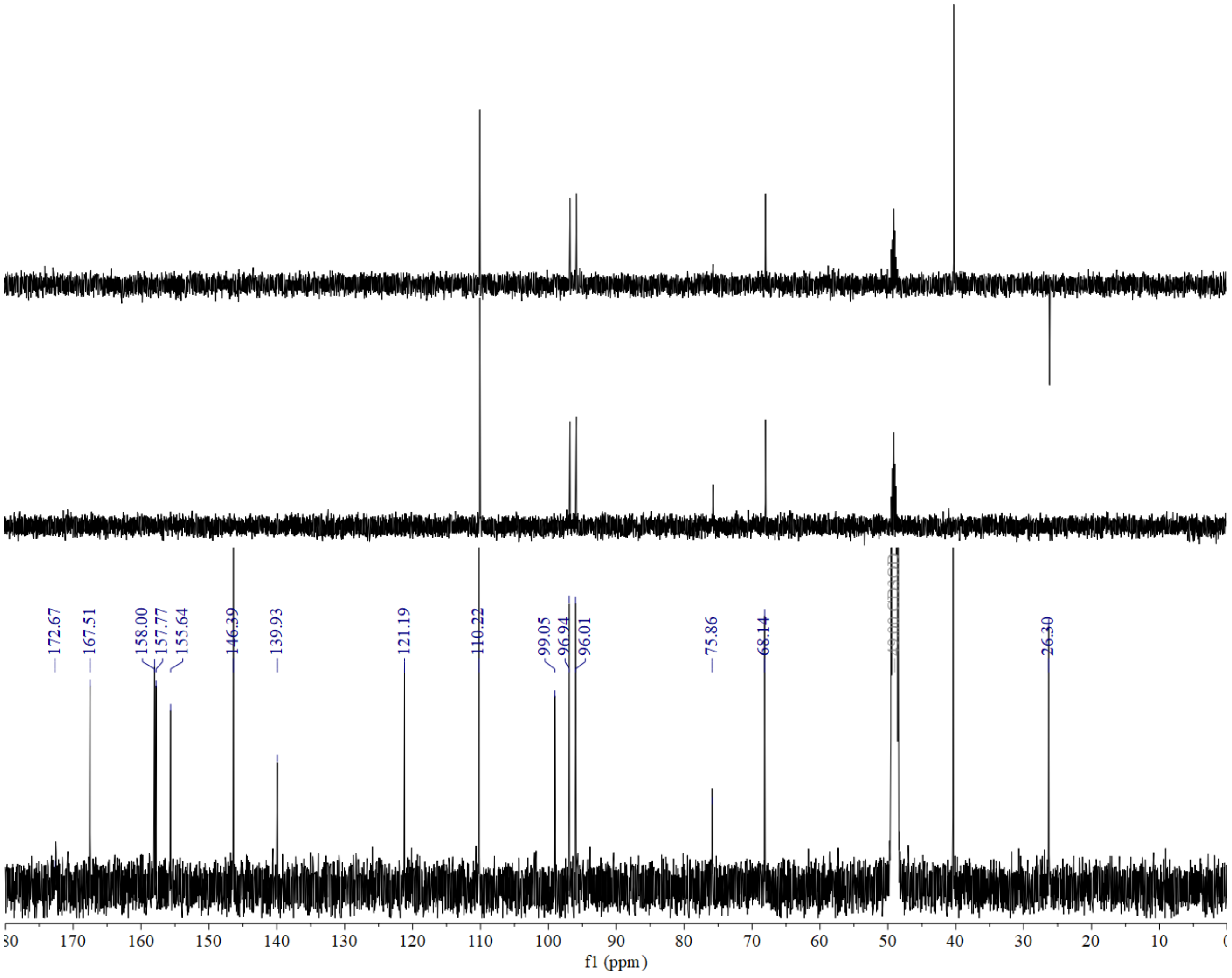

Figure S20. HSQC spectrum of compound **3** in CD_3OD

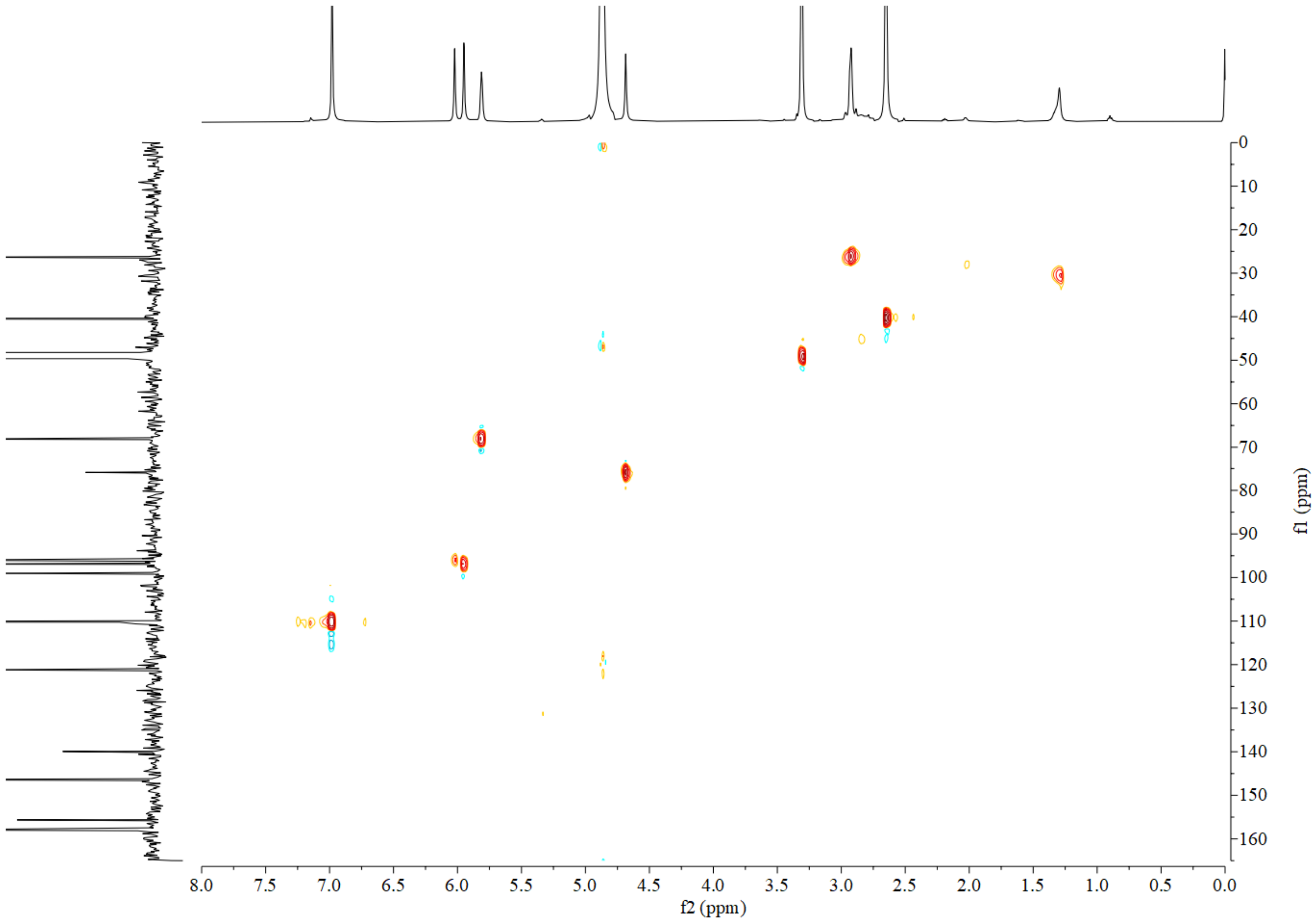

Figure S21. HMBC spectrum of compound **3** in CD_3OD

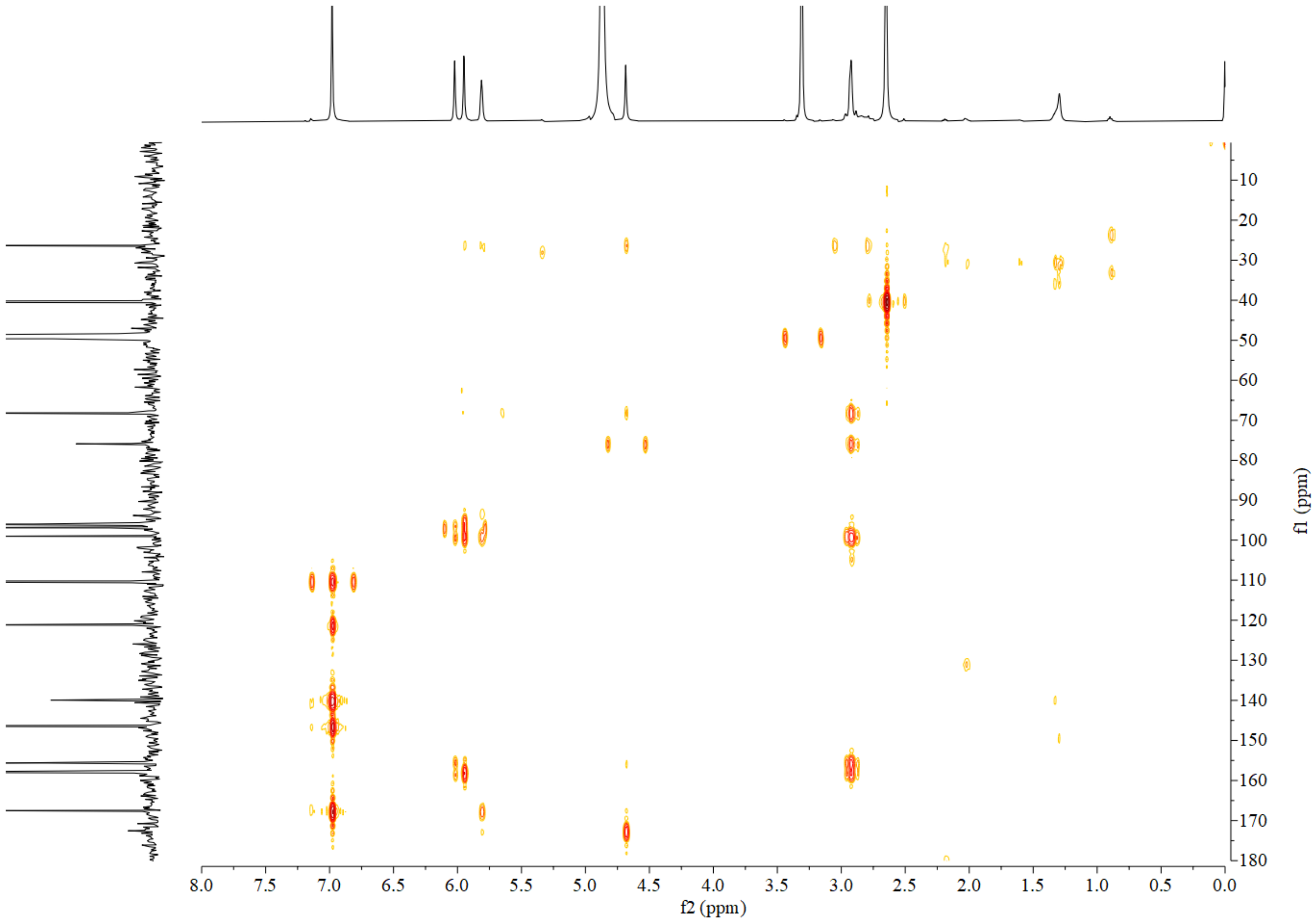

Figure S22. COSY spectrum of compound **3** in CD_3OD

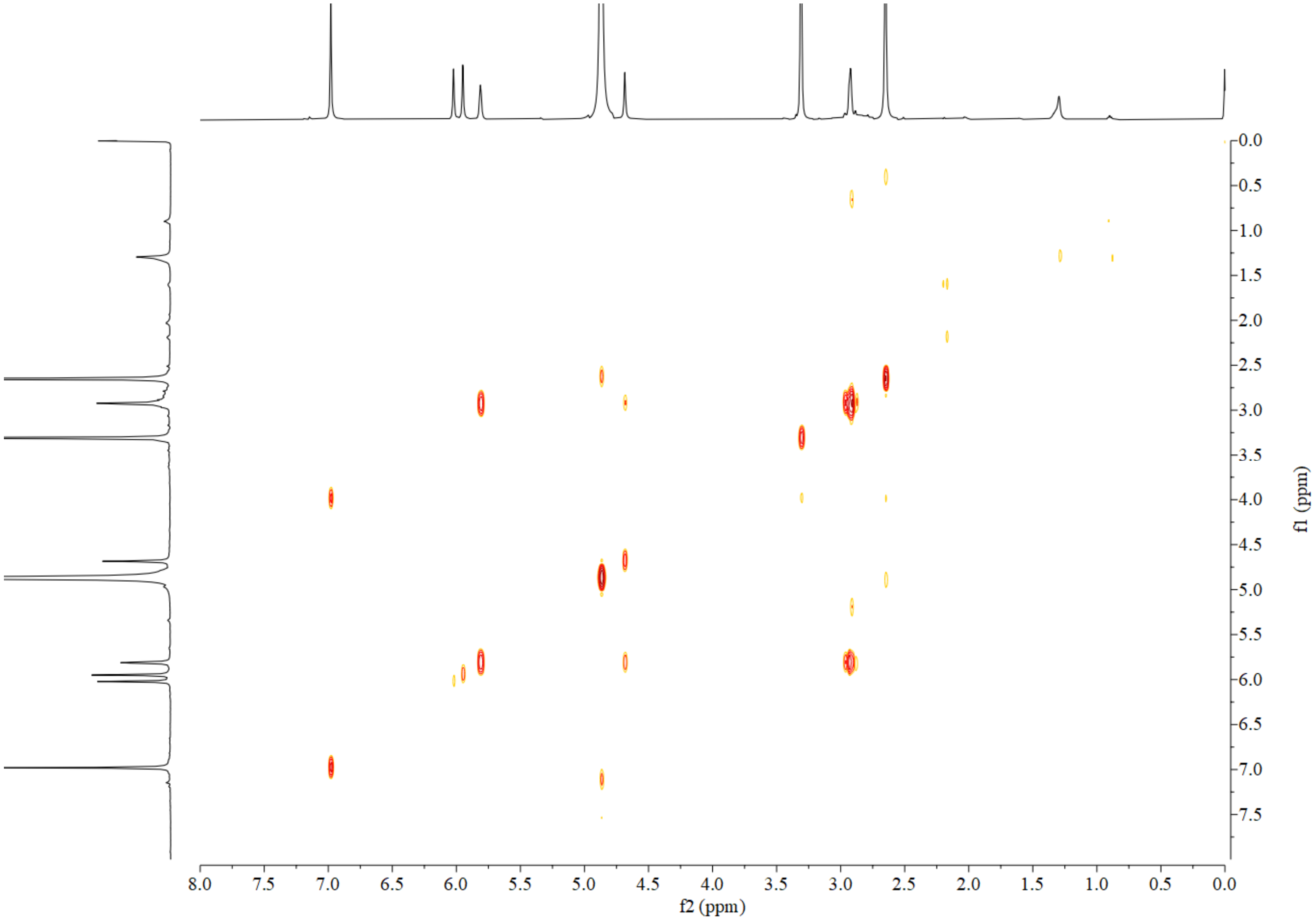

Figure S23. ROESY spectrum of compound **3** in CD_3OD

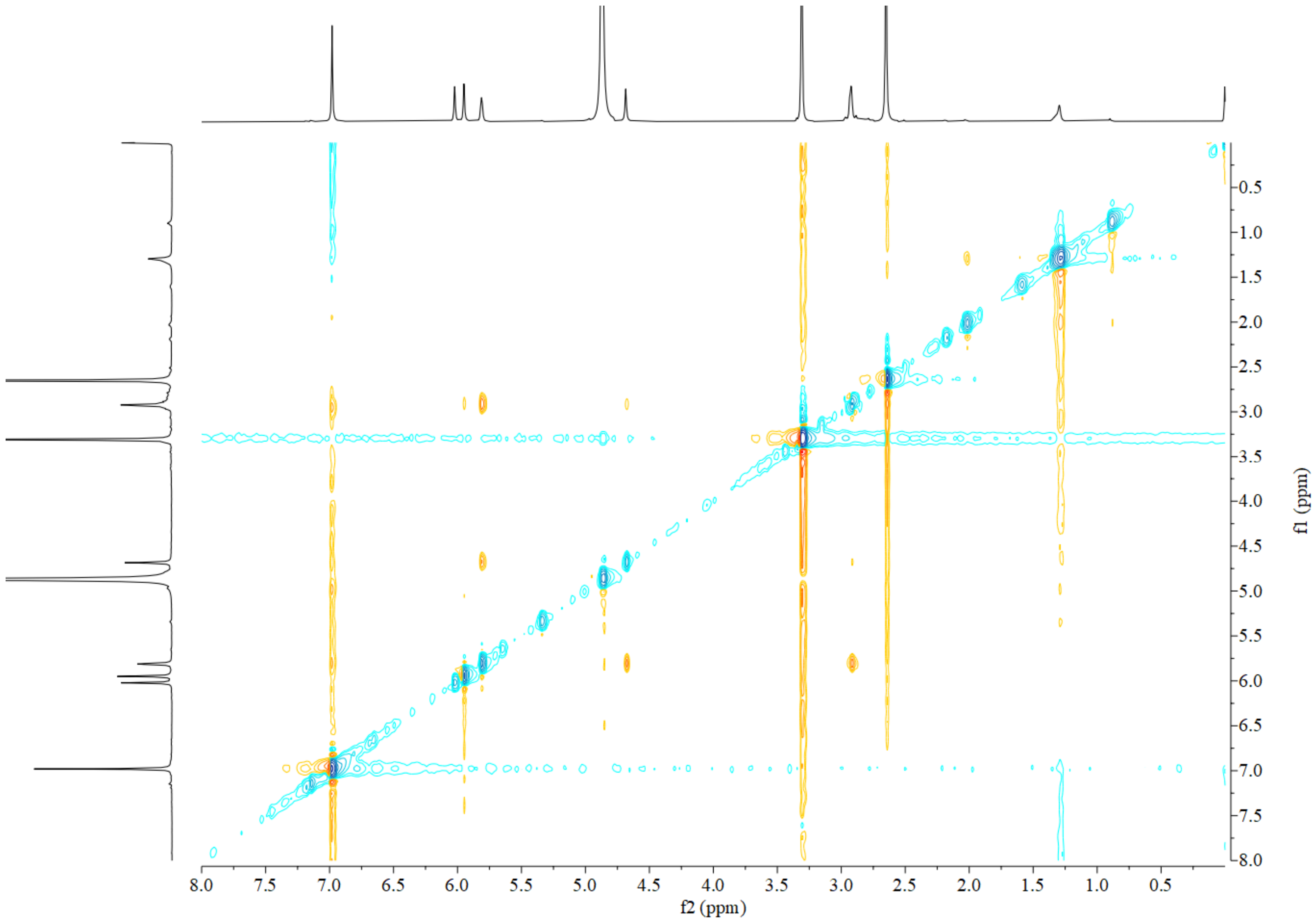

Figure S24.   HRESI-MS spectrum of compound **3**

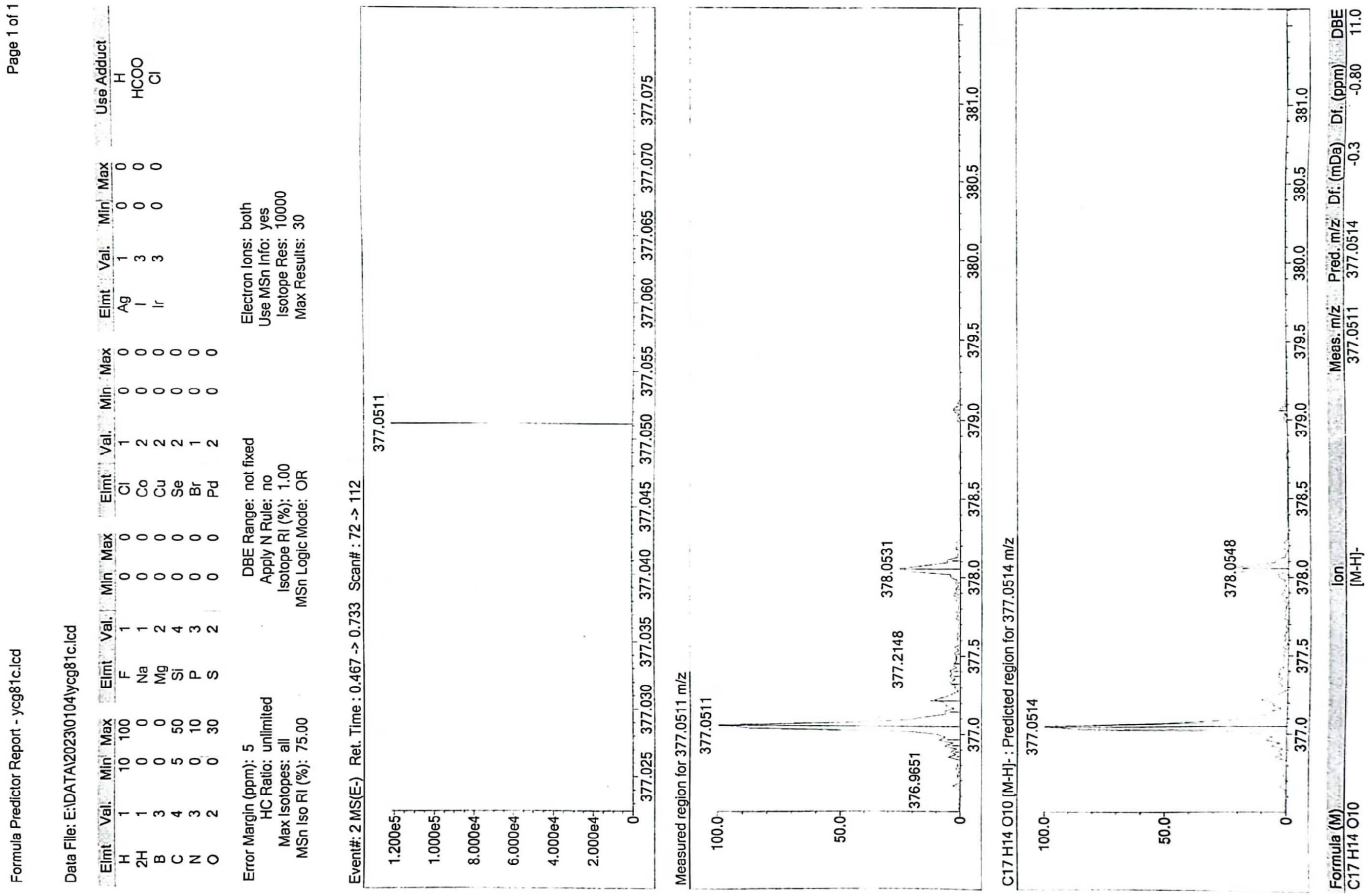

Figure S25. CD and UV spectra of compound **3** in MeOH

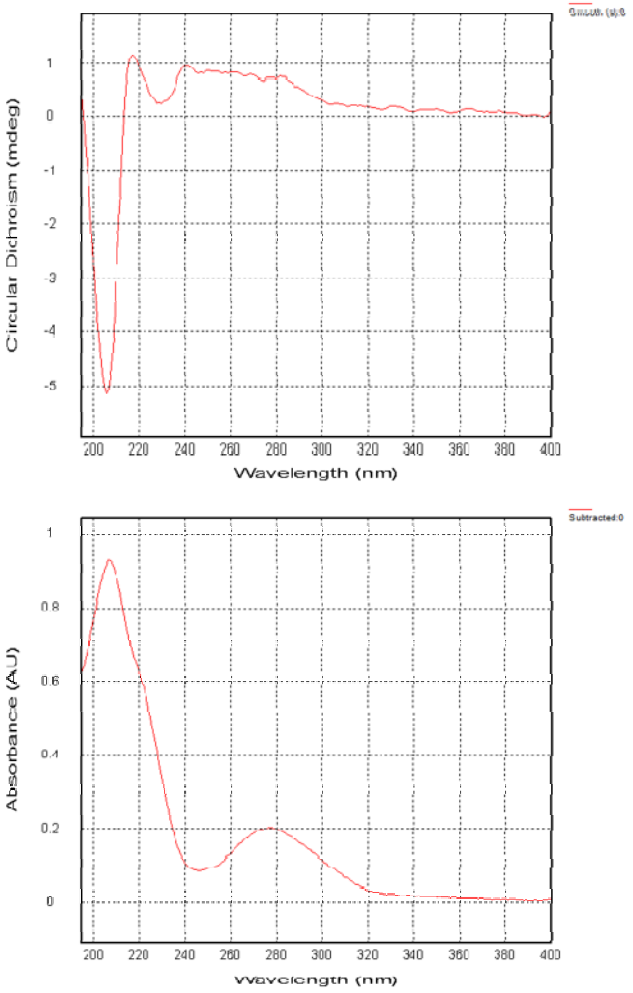

File: YCG81C-1mm(195-400)23011310.dsx
ProBinaryX
Attributes :
- Time Stamp :Fri Jan 13 16:07:46 2023

- File ID : {12DE714C-FF47-4d11-8127-8A6E3C31486E}
- Is CFR Compliant : false
- Original unaltered data

Remarks:
- User: CD
- Date: 2023/01/13
- Instrument: 0547
- DetectorType: LAAPD
- DichOS Calibration Correction Curve: 0547/2
- HV (CDDC channel): 0 v
- Time per point: 1 s
- Description: Sample 1
- Concentration: 0.0679mg/mLMeOH
- Pathlength: 1 mm
- Temperature: 20°C

Settings:
- HV
- Time-per-point: 1s (25us x 40000)
- SE
- Wavelength: 195nm - 400nm
- Step Size: 1nm
- Bandwidth: 1nm

Figure S26. OR of compound **3** in MeOH

Rudolph Research Analytical

This sample was measured on an Autopol VI, Serial #91058
Manufactured by Rudolph Research Analytical, Hackettstown, NJ, USA.

Measurement Date : Tuesday, 10-JAN-2023

Set Temperature : 20.0

Time Delay : Disabled

Delay between Measurement : Disabled

n	Average	Std.Dev.	% RSD	Maximum	Minimum
5	-39.18	0.73	-1.86	-38.14	-40.21

S.No	Sample ID	Time	Result	Scale	OR °Arc	WLG.nm	Lg.mm	Conc.g/100ml	Temp.
1	YCG81C	03:49:16 PM	-40.21	SR	-0.039	589	100.00	0.097	20.2
2	YCG81C	03:49:22 PM	-39.18	SR	-0.038	589	100.00	0.097	20.2
3	YCG81C	03:49:28 PM	-39.18	SR	-0.038	589	100.00	0.097	20.1
4	YCG81C	03:49:35 PM	-39.18	SR	-0.038	589	100.00	0.097	20.1
5	YCG81C	03:49:41 PM	-38.14	SR	-0.037	589	100.00	0.097	20.1

Figure S27. ¹H NMR spectrum of compound **4** in CD_3OD

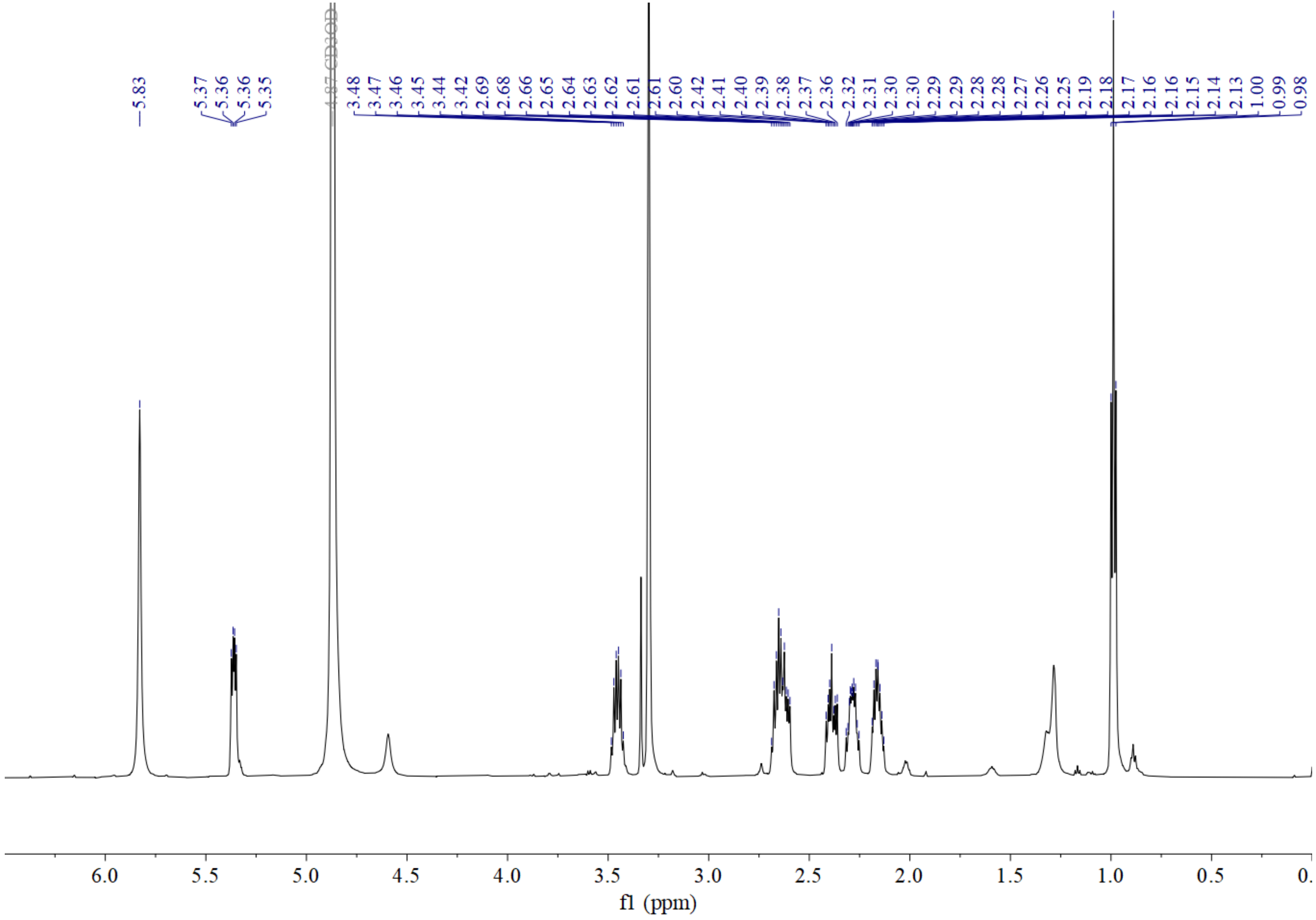

Figure S28. ^{13}C NMR spectrum of compound **4** in CD_3OD

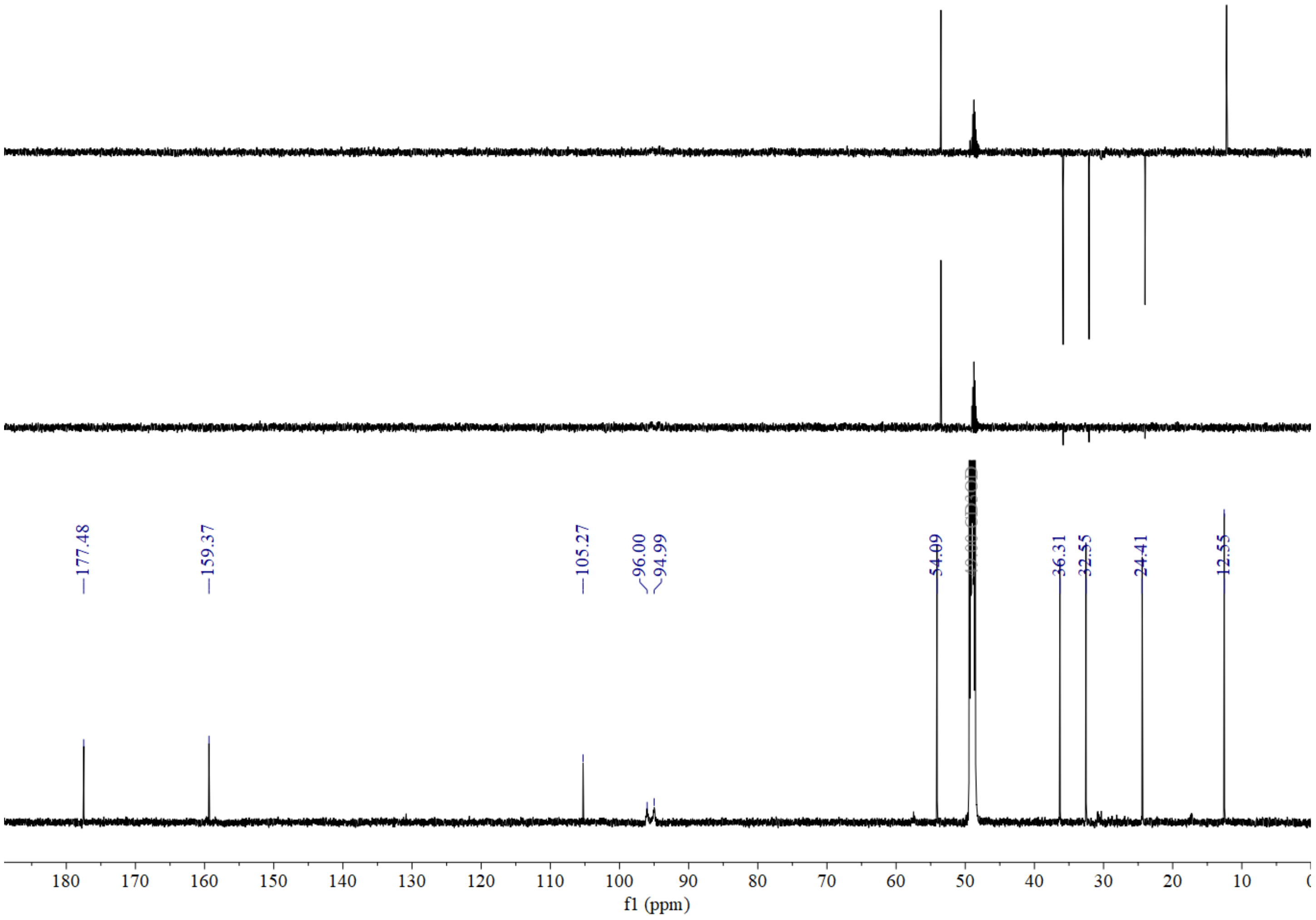

Figure S29. HSQC spectrum of compound **4** in CD_3OD

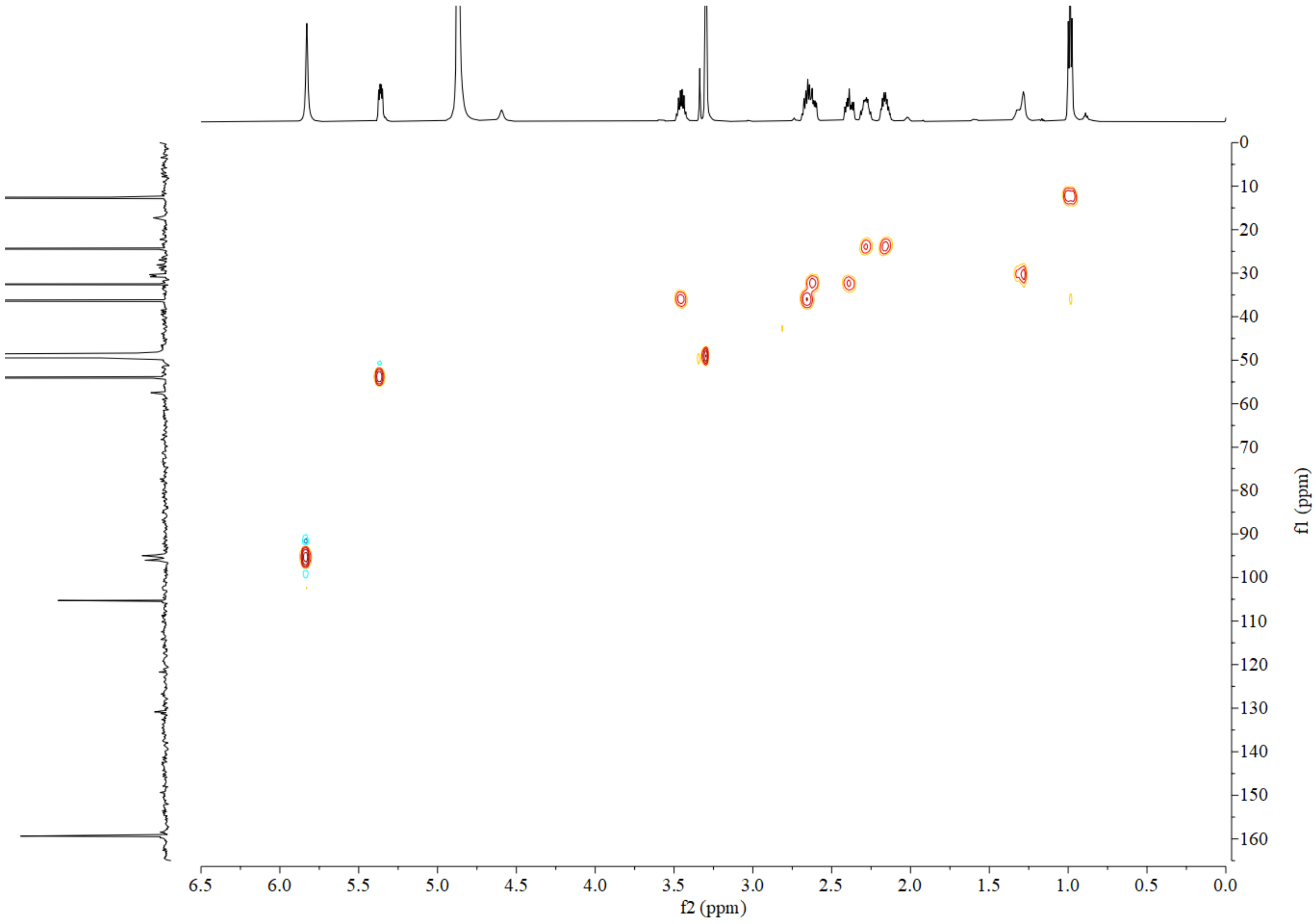

Figure S30. HMBC spectrum of compound **4** in CD_3OD

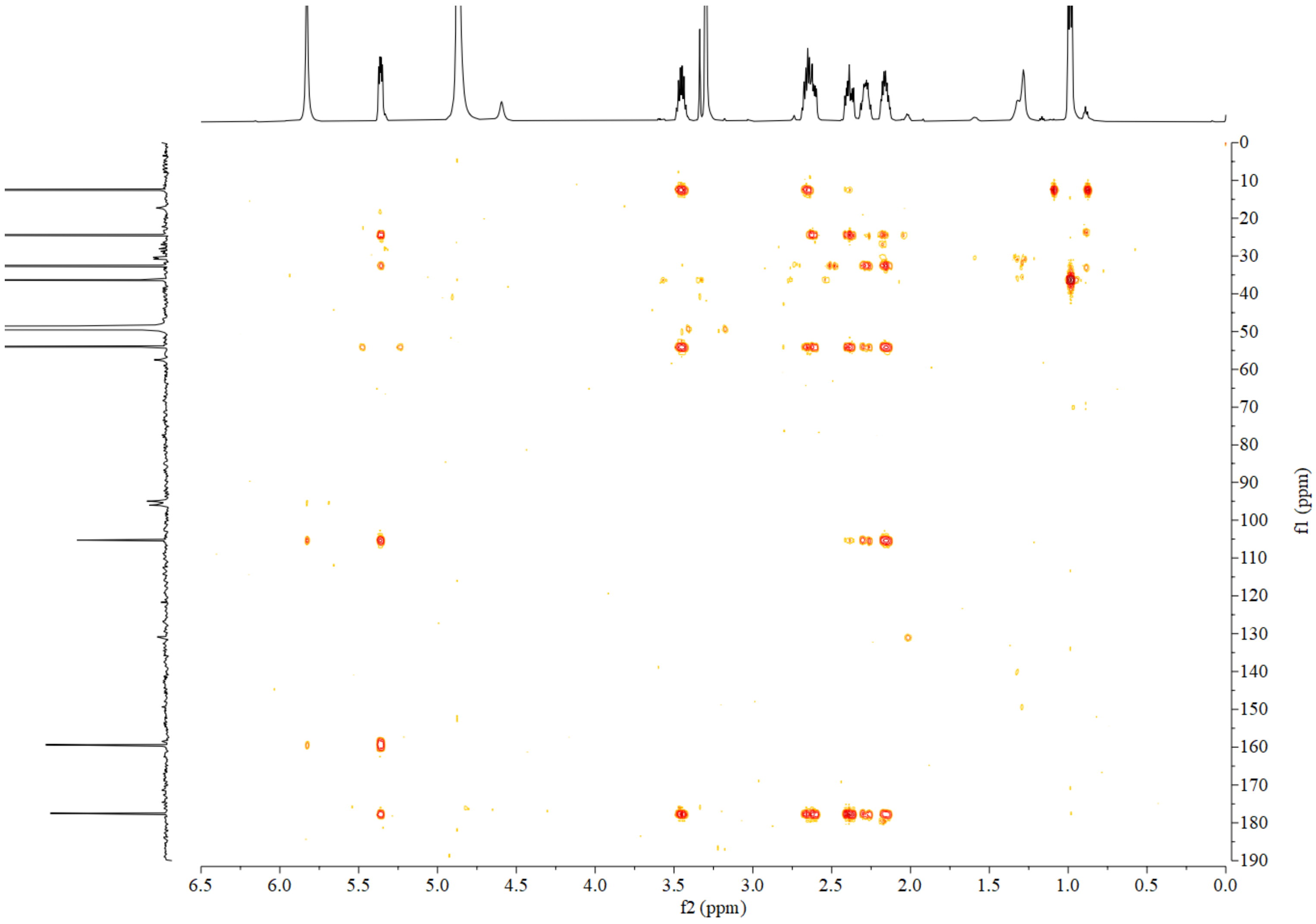

Figure S31. COSY spectrum of compound **4** in CD_3OD

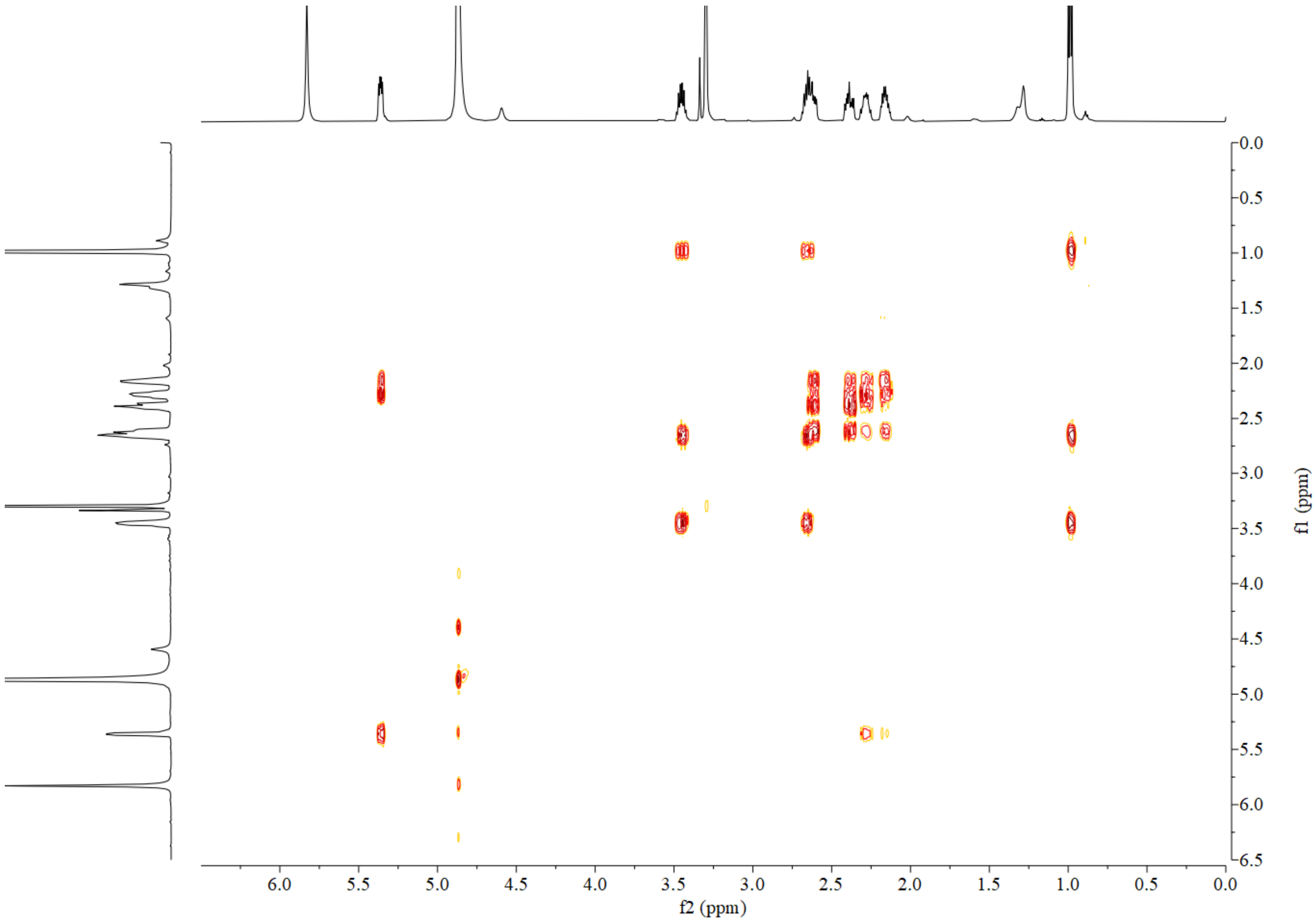

Figure S32. ROESY spectrum of compound **4** in CD_3OD

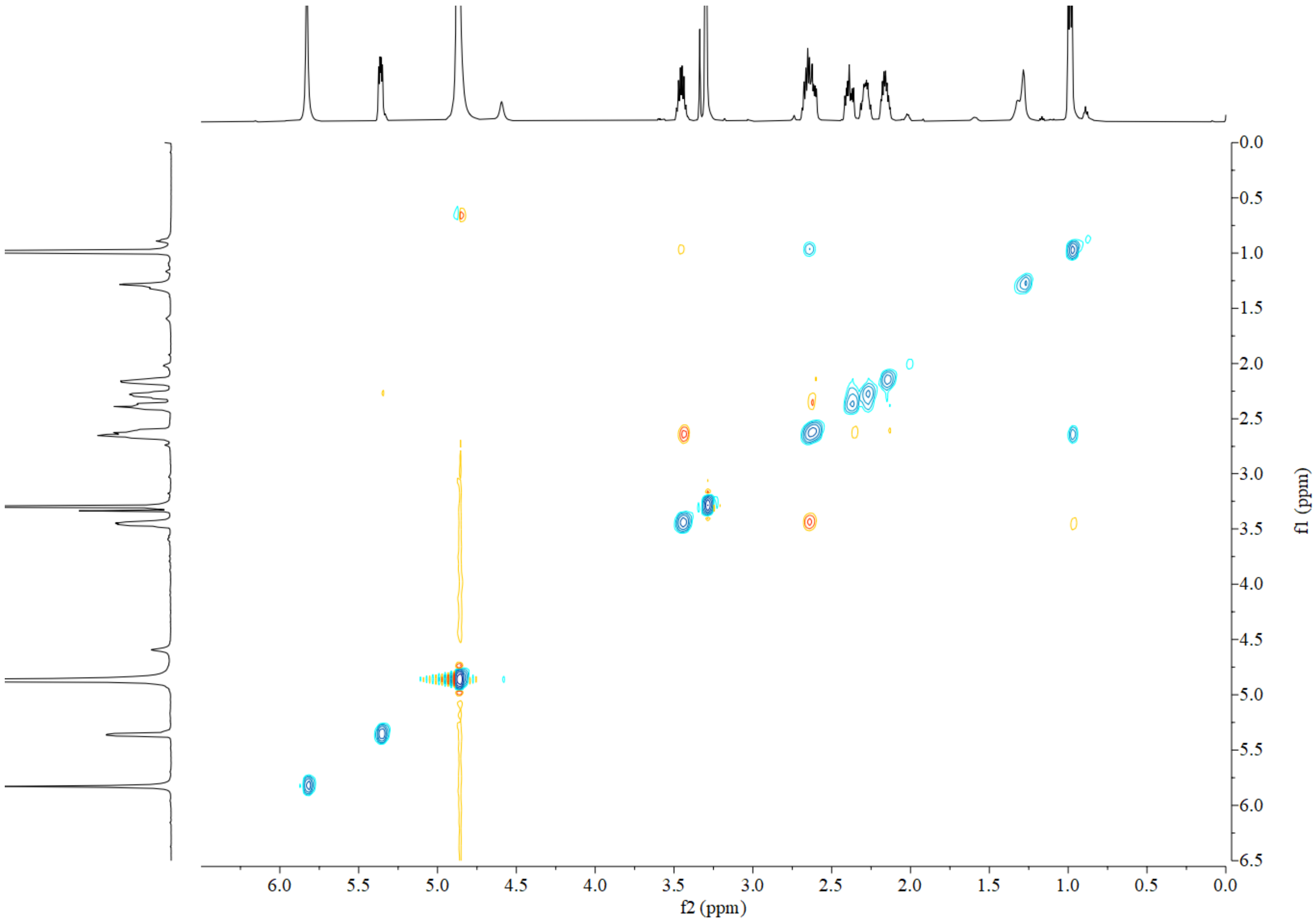

Figure S33. HRESI-MS spectrum of compound **4**

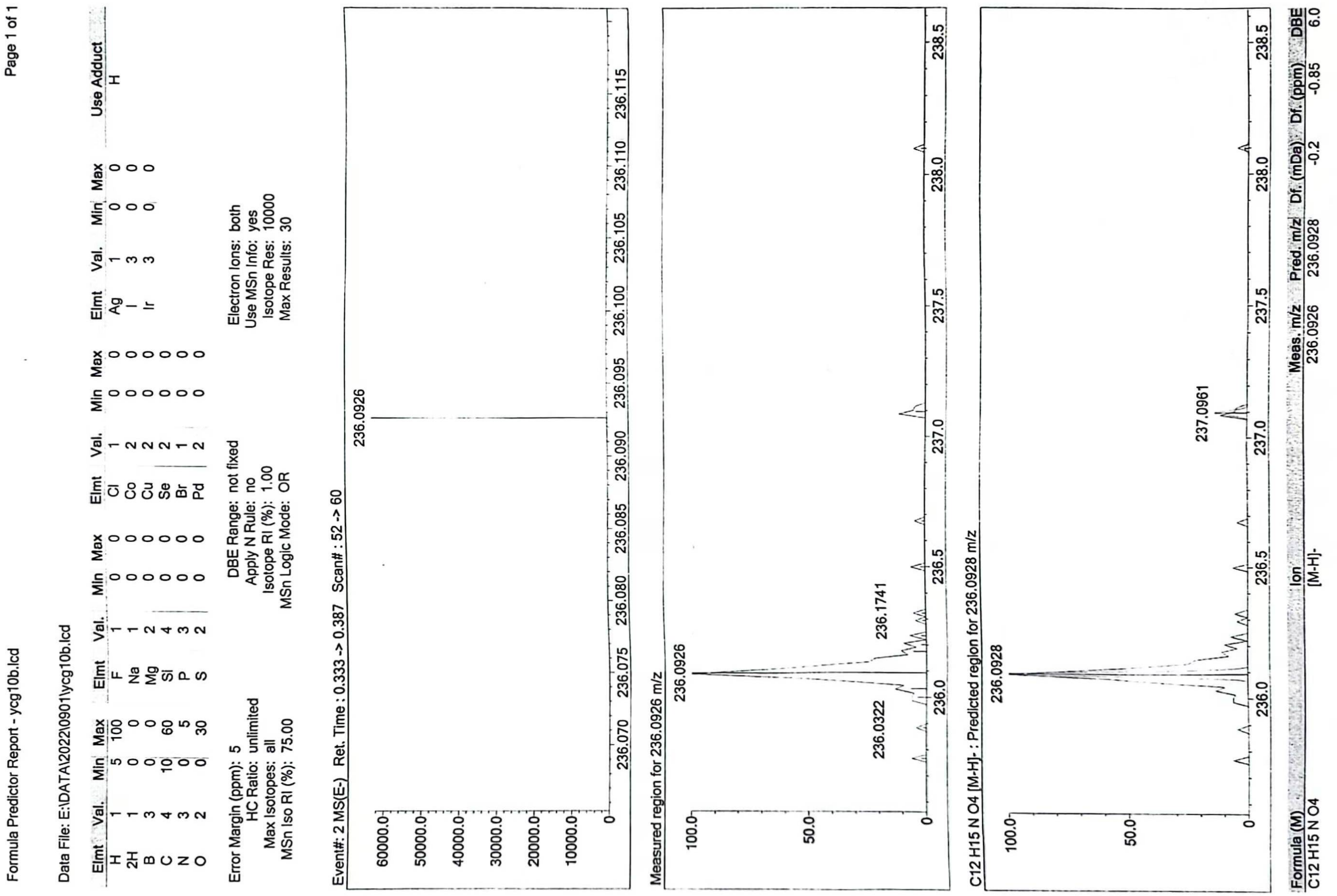

Figure S34. CD and UV spectra of compound **4** in MeOH

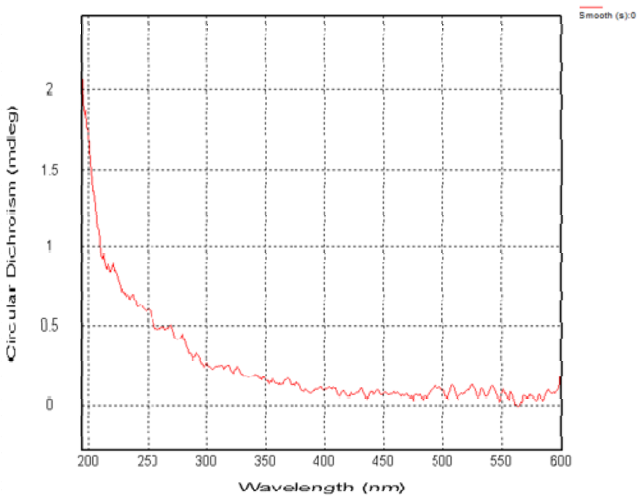

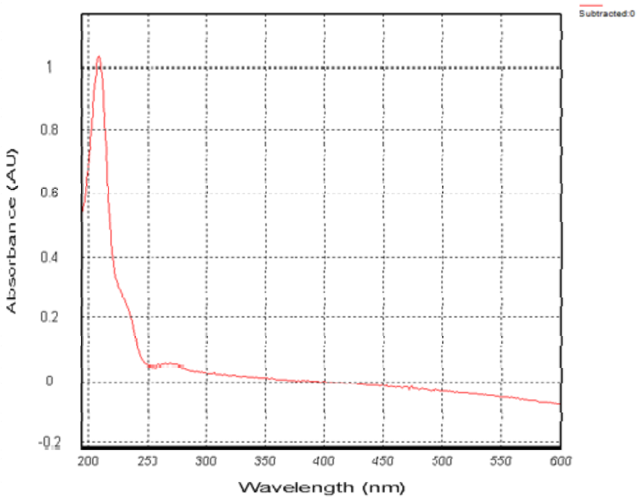

File: YCG10B-1mm(195-600)22090209.dsx
ProBinaryX
Attributes :
- Time Stamp :Fri Sep 02 16:19:18 2022

- File ID : {4D8EFF47-CC76-4ab9-8443-486DF7BFCFD6}
- Is CFR Compliant : false
- Original data has not been modified.

Remarks:
- User: CD
- Date: 2022/09/02
- Instrument: 0547
- DetectorType: LAAPD
- DichOS Calibration Correction Curve: 0547/2
- HV (CDDC channel): 0 v
- Time per point: 1 s
- Description: Sample 1
- Concentration: 0.0528 mg/mL MeOH
- Pathlength: 1 mm
- Temperature: 20°C

Settings:
- Time-per-point: 1s (25us x 40000)
- SE
- Wavelength: 195nm - 600nm
- Step Size: 1nm
- Bandwidth: 1nm

Figure S35. OR of compound **4** in MeOH

Rudolph Research Analytical

This sample was measured on an Autopol VI, Serial #91058
Manufactured by Rudolph Research Analytical, Hackettstown, NJ, USA.

Measurement Date : Thursday, 01-SEP-2022

Set Temperature : OFF

Time Delay : Disabled

Delay between Measurement : Disabled

n	Average	Std.Dev.	% RSD	Maximum	Minimum
5	-23.40	1.14	-4.87	-22.00	-25.00

S.No	Sample ID	Time	Result	Scale	OR °Arc	WLG.nm	Lg.mm	Conc.g/100ml	Temp.
1	YCB10B	04:20:54 PM	-23.00	SR	-0.023	589	100.00	0.100	23.3
2	YCB10B	04:21:00 PM	-22.00	SR	-0.022	589	100.00	0.100	23.3
3	YCB10B	04:21:06 PM	-25.00	SR	-0.025	589	100.00	0.100	23.3
4	YCB10B	04:21:13 PM	-24.00	SR	-0.024	589	100.00	0.100	23.3
5	YCB10B	04:21:19 PM	-23.00	SR	-0.023	589	100.00	0.100	23.3

Figure S36. ^{1}H NMR spectrum of compound **6** in CD_3OD

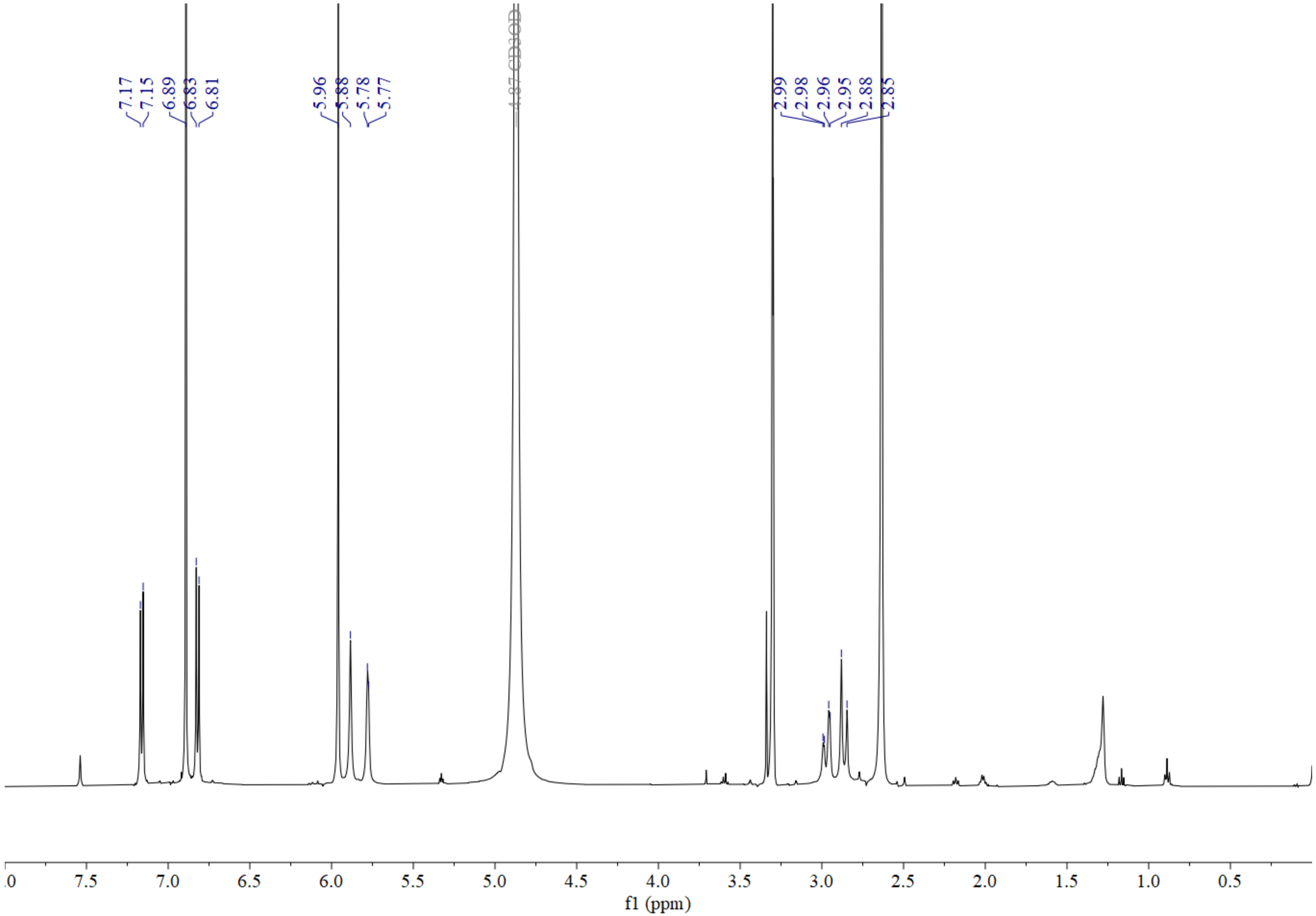

Figure S37. ^{13}C NMR spectrum of compound **6** in CD_3OD

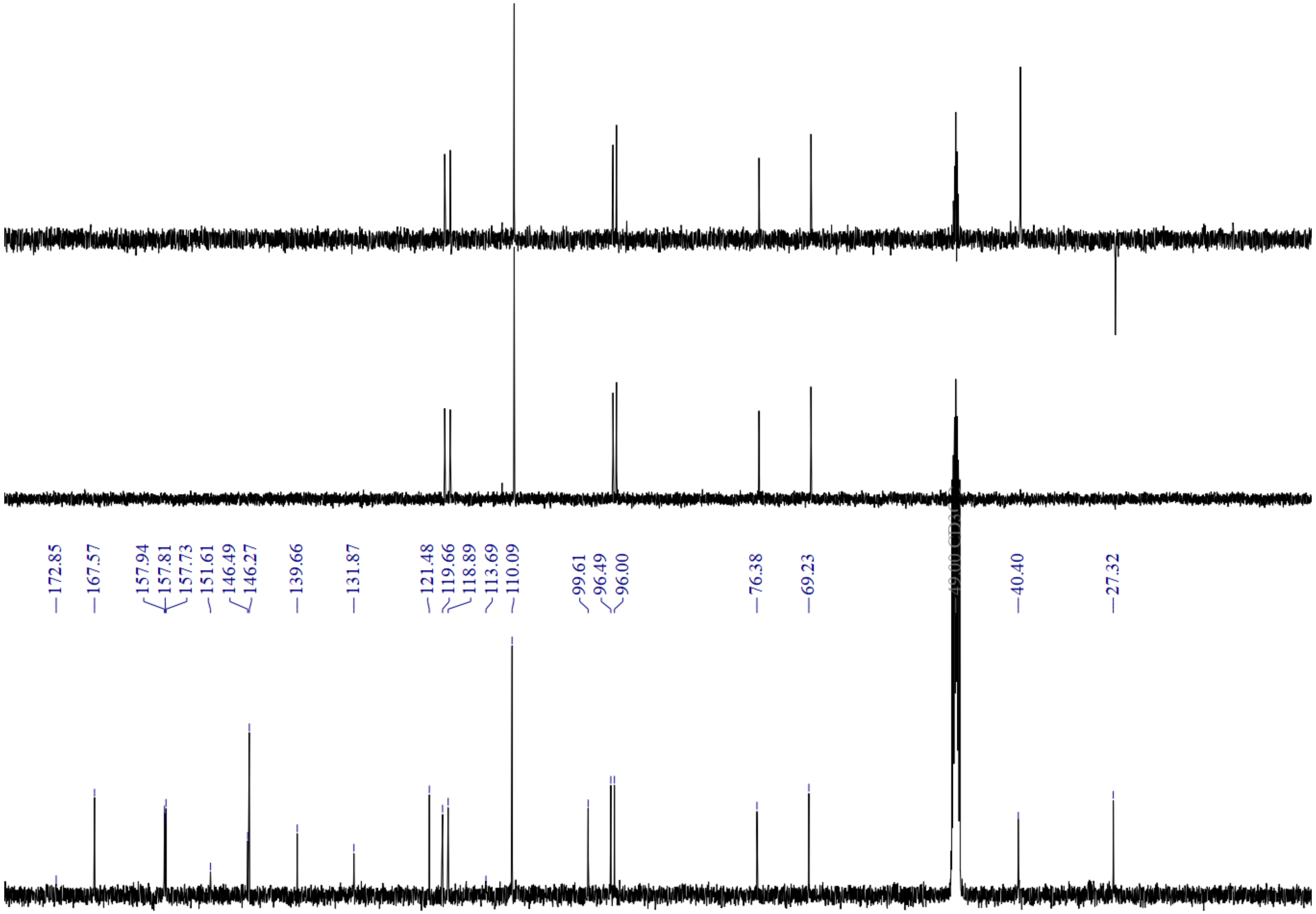

Figure S38. Possible formation mechanisms of compounds **1** and **2**

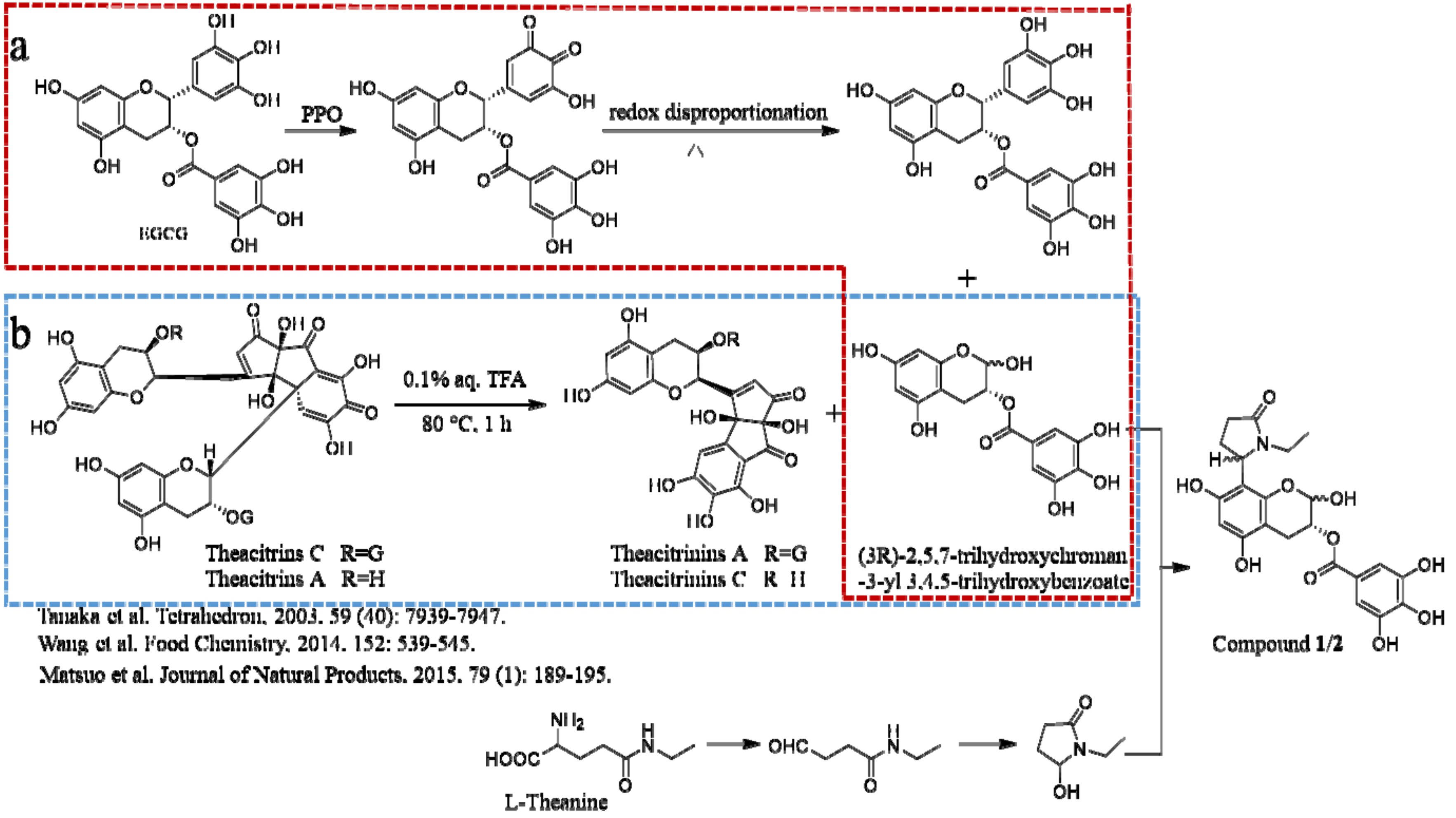

Table S1. ^{13}C and ^{1}H NMR spectroscopic data of compound **1** in CD_3OD

No.	**1a** (2*R*,3*R*,5''*R*)		**1b** (2*S*,3*R*,5''*R*)	
	δ_C, type	δ_H (*J* in Hz)	δ_C, type	δ_H (*J* in Hz)
2	91.1, d	5.42, br.s	91.6, d	5.48, br.s
3	68.1, d	5.07, d, *J* = 3.4 Hz	68.4, d	5.13, d, *J* = 2.7 Hz
4	21.1, t	a 2.96–2.83, m<br>b 2.78, d, *J* = 17.4 Hz	21.1, t	a 2.96–2.83, m<br>b 2.78, d, *J* = 17.4 Hz
5	157.0, s		157.0, s	
6	96.0, d	6.01, s	96.9, d	5.94, s
7	156.1, s		156.1, s	
8	106.0, s		106.0, s	
9	153.0, s		153.0, s	
10	100.1, s		100.1, s	
1'	121.2, s		121.3, s	
2'	110.2, d	6.88, s	110.3, d	7.06, s
3'	146.4, s		146.5, s	
4'	139.9, s		140.0, s	
5'	146.4, s		146.5, s	
6'	110.2, d	6.88, s	110.3, d	7.06, s
7'	167.9, s		167.6, s	
2''	177.6, s		177.6, s	
3''	32.6, t	a 2.64–2.56, m<br>b 2.44–2.35, m	32.6, t	a 2.64–2.56, m<br>b 2.44–2.35, m
4''	24.6, t	a 2.29, dtd, *J* = 16.0, 9.5, 5.2 Hz<br>b 2.16, dq, *J* = 16.0, 5.6 Hz	24.7, t	a 2.29, dtd, *J* = 16.0, 9.5, 5.2 Hz<br>b 2.16, dq, *J* = 16.0, 5.6 Hz
5''	54.3, d	5.38, dd, *J* = 9.5, 5.6 Hz	54.1, d	5.38, dd, *J* = 9.5, 5.6 Hz
6''	36.6, t	a 3.35–3.30, m<br>b 2.66, m	36.4, t	a 3.35–3.30, m<br>b 2.66, m
7''	12.7, q	0.98, t, *J* = 7.2 Hz	12.6, q	0.91, t, *J* = 7.4 Hz

Table S2. ^{13}C and ^{1}H NMR spectroscopic data of compound **2** in CD_3OD

No.	**2a** (2*R*,3*R*,5''*S*)		**2b** (2*S*,3*R*,5''*S*)	
	δ_C, type	δ_H (*J* in Hz)	δ_C, type	δ_H (*J* in Hz)
2	91.2, d	5.60–5.48, m	91.2, d	5.40, d, *J* = 4.3 Hz
3	68.1, d	5.14, dd, *J* = 4.5, 2.6 Hz	69.5, d	5.09, m
4	21.3, t	a 2.99–2.86, br.d, *J* = 17.2 Hz	21.3, t	a 2.99–2.86, br.d, *J* = 17.2 Hz
		b 2.85–2.73, br.d, *J* = 17.2 Hz		b 2.85–2.73, br.d, *J* = 17.2 Hz
5	156.8, s		156.8, s	
6	96.1, d	6.02, s	96.9, d	5.96, s
7	157.0, s		157.0, s	
8	105.9, s		105.9, s	
9	153.0, s		153.0, s	
10	99.1, s		99.1, s	
1'	121.3, s		121.3, s	
2'	110.1, d	6.93, s	110.3, d	7.09, s
3'	146.4, s		146.5, s	
4'	140.0, s		140.0, s	
5'	146.4, s		146.5, s	
6'	110.1, d	6.93, s	110.3, d	7.09, s
7'	167.7, s		167.7, s	
2''	177.6 s		178.0, s	
3''	32.6, t	a 2.68, dd, *J* = 15.3, 8.5 Hz	32.6, t	a 2.68, dd, *J* = 15.3, 8.5 Hz
		b 2.41, t, *J* = 9.1 Hz		b 2.41, t, *J* = 9.1 Hz
4''	24.5, t	a 2.35–2.24, m	24.5, t	a 2.35–2.24, m
		b 2.18, dd, *J* = 9.4, 6.0 Hz		b 2.18, dd, *J* = 9.4, 6.0 Hz
5''	54.0, d	5.42, dd, *J* = 9.4, 5.2 Hz	53.8, d	5.42, dd, *J* = 9.4, 5.2 Hz
6''	36.3, t	a 3.56–3.40, m	36.5, t	a 3.56–3.40, m
		b 2.62, dq, *J* = 14.2, 7.6 Hz		b 2.62, dq, *J* = 14.2, 7.3 Hz
7''	12.5, q	1.00, t, *J* = 7.6 Hz	12.6, q	0.89, t, *J* = 7.3 Hz

Table S3. ^{13}C and ^{1}H NMR spectroscopic data of compounds **3** and **6** in CD_3OD

No.	**3**		**6**	
	δ_C, type	δ_H (J in Hz)	δ_C, type	δ_H (J in Hz)
2	75.9, d	4.69, s	76.4, d	5.88, s
3	68.1, d	5.81, br.s	69.2, d	5.78, d, J = 3.9 Hz
4	26.3, t	2.99–2.87, m	27.3, t	2.86, d, J = 17.2Hz
				2.97, dd, J = 17.6, 4.3 Hz
5	157.8, s		157.7, s	
6	96.9, d	5.95, d, J = 2.4 Hz	96.5, d	5.96, d, J = 2.1 Hz
7	158.0, s		158.0, s	
8	96.0, d	6.02, d, J = 2.4 Hz	96.0, d	5.96, d, J = 2.1 Hz
9	155.6, s		157.8, s	
10	99.0, s		99.6, s	
1'	121.2, s		121.5, s	
2'	110.2, d	6.98, s	110.1, d	6.89, s
3'	146.4, s		146.3, s	
4'	140.0, s		139.7, s	
5'	146.4, s		146.3, s	
6'	110.2, d	6.98, s	110.1, d	6.89, s
7'	167.5, s		167.6, s	
COOH	172.7, s		172.8, s	
1''			131.9, s	
2''			113.7, s	
3''			151.6, s	
4''			146.5, s	
5''			118.9, d	6.82, d, J = 8.4 Hz
6''			119.7, d	7.16, d, J = 8.4 Hz

Table S4. ^{13}C and ^{1}H NMR spectroscopic data of compound **4** in CD_3OD

			**4**		
No.	δ_C, type	δ_H (*J* in Hz)	No.	δ_C, type	δ_H (*J* in Hz)
1	105.3, s		2'	177.5, s	
2	159.4, s		3'	32.6, t	a 2.62, m
3	96.0, d	5.83, s			b 2.39, ddd, *J* =16.6, 10.7, 6.0 Hz
4	159.4, s		4'	24.4, t	a 2.33–2.24, m
5	95.0, d	5.83, s			b 2.16, dq, *J* = 16.6, 5.6 Hz
6	159.4, s		5'	54.1, d	5.36, dd, *J* = 9.6, 5.6 Hz
			6'	36.3, t	a 3.45, dq, *J* =14.5, 7.2 Hz
					b 2.65, ddd, *J* =14.5, 7.0, 6.8 Hz
			7'	12.6, q	0.99, t, *J* = 7.2 Hz
